# Supplementary material for: Mechanisms of drug interactions between translation-inhibiting antibiotics
Source: Nat Commun. 2020 Aug 11;11:4013. doi: 10.1038/s41467-020-17734-z (PMC7421507; doi:10.1038/s41467-020-17734-z)
Supplement: Supplementary file 1 — Supplementary Information [file 41467_2020_17734_MOESM1_ESM.pdf]

Supplementary Information

Mechanisms of drug interactions between translation-inhibiting antibiotics

Kavčič *et al.*

Supplementary Methods

Quantification of the drug interaction type and bottleneck dependency

Loewe interaction score

To quantify the drug interaction between a pair of antibiotics, we defined the Loewe interaction score as

$$\mathrm{LI} = \log\left(\frac{\int g(x_1, x_2)\mathrm{d}x_1\mathrm{d}x_2}{\int g_{\mathrm{add}}(x_1, x_2)\mathrm{d}x_1\mathrm{d}x_2}\right), \quad (1)$$

where $g(x_1, x_2)$ and $g_{\mathrm{add}}(x_1, x_2)$ are the measured and the predicted additive dose-response surfaces over a 2D concentration field (x_1, x_2), respectively. The LI score is a log-transformed ratio of volumes underneath the dose-response surfaces. It is positive for antagonistic and suppressive interactions, zero for perfectly additive, and negative for synergistic interactions. To avoid imposing arbitrary bounds for classifying a measured interaction as synergistic or antagonistic/suppressive (rather than additive), we performed smooth bootstrapping on a set of ideal additive response surfaces to establish a distribution of interaction indices expected for perfectly additive but noisy surfaces. To achieve this, we generated additive dose-response surfaces for drugs with a Hill steepness parameter n between 1.8 and 6.6 (obtained as 10% and 90% percentiles of the distribution of steepness parameters for measured dose-response curves). We estimated the variabilities of measurements σ_{v} from data from eight replicated dose-response curves with seven replicates per data point and fitting errors σ_{f} from the slope of all growth rate fits. Both error and variability distributions were well described by log-normal distributions. For each point on the generated surfaces, we added Gaussian noise with standard deviation given as $\sqrt{\sigma_{\mathrm{v}}^2 + \sigma_{\mathrm{f}}^2}$, where both σ_{v} and σ_{f} were drawn from respective log-normal distributions. We calculated the LI score for 2,000 response surfaces and obtained the distribution shown in Supplementary Figure 1d. We determined boundaries separating synergistic and antagonistic LI scores (b_{lower} and b_{upper}, respectively) from additive interval as Bonferroni-corrected percentiles (for $5\%/28 \approx 0.18\%$ and $100\% - (5\%/28) \approx 99.82\%$) of the bootstrapped distribution (Supplementary Figure 1d). Mean LI scores for measured response surfaces falling outside of the interval with these boundaries were classified as synergistic or antagonistic; otherwise, the interaction was classified as additive.

Bottleneck dependency score

Similar to LI, the bottleneck dependency score BD is an integrative quantity that concisely reports on the response-averaged deviation from independence. To calculate this score, the antibiotic and inducer concentrations are first converted into corresponding responses using the induction- and antibiotic dose-response curves (Supplementary Figure 3). Mathematically, this means that $r_{\mathrm{x}} = y(c)$ and $r_{\mathrm{y}} = g(b)$ for antibiotic and inducer, respectively. In response space, the null-expectation is independence, i.e., the expected response is a product of individual responses. Thus, we define the BD score as

$$\mathrm{BD} = \log\left(\frac{\int r(r_{\mathrm{x}}, r_{\mathrm{y}})\mathrm{d}r_{\mathrm{x}}\mathrm{d}r_{\mathrm{y}}}{\int r_{\mathrm{x}} r_{\mathrm{y}}\mathrm{d}r_{\mathrm{x}}\mathrm{d}r_{\mathrm{y}}}\right). \quad (2)$$

This score is zero when the two perturbations (bottleneck and antibiotic) are independent; it is positive or negative for alleviation and aggravation, respectively. As for the LI score, we evaluated the independence interval of BD scores by bootstrapping the BD score for independent surfaces at given induction and antibiotic dose-response curves. Evaluating the percentiles of such null-distributions gave the boundaries for evaluation of the type of deviation from independence (alleviation or aggravation).

Growth law-based biophysical model

Single antibiotic The mathematical model used for predicting bacterial growth in the presence of antibiotic combination is an extension of the model presented in Ref. [1]. An in-depth analysis of the model is presented

in Ref. [2]. In brief, the model captures the crucial processes of antibiotic binding and transport as well as physiological constraints. We briefly summarize the results for a single antibiotic and its main ingredients. The growth laws are given as

$$r_u = \lambda/\kappa_t + r_{min}, \tag{3}$$

and

$$r_{tot} = r_u + r_b = r_{max} - \lambda \Delta r \left(\frac{1}{\lambda_0} - \frac{1}{\kappa_t \Delta r} \right), \tag{4}$$

where r_u, r_b, and r_{tot} are concentrations of unbound, bound, and total ribosomes. The constants $\kappa_t \approx 0.06\,\mu\text{M}^{-1}\text{h}^{-1}$, $r_{min} = 19.3\,\mu$M, $r_{max} = 65.8\,\mu$M and $\Delta r = r_{max} - r_{min} = 46.5\,\mu$M were experimentally determined in Refs. [3, 1]. Transport of antibiotic is captured by the average flux as $J(a_{ex}, a) = p_{in} a_{ex} - p_{out} a$, where p_{in} and p_{out} are influx and efflux rates, respectively, and a and a_{ex} are the intracellular and external antibiotic concentration, respectively. The kinetics of binding of the antibiotic to the ribosome is given as $f(r_u, r_b, a) = -k_{on} a (r_u - r_{min}) + k_{off} r_b$, where k_{on} and k_{off} are binding and unbinding rates, respectively, and $K_D = k_{off}/k_{on}$. The fraction of inactive ribosomes r_{min} is assumed not to bind antibiotics [1]. The following system of ordinary differential equations (ODEs) describes the kinetics of the system:

$$\frac{\mathrm{d}a}{\mathrm{d}t} = -\lambda a + f(r_u, r_b, a) + J(a_{ex}, a), \tag{5}$$

$$\frac{\mathrm{d}r_u}{\mathrm{d}t} = -\lambda r_u + f(r_u, r_b, a) + s(\lambda), \tag{6}$$

$$\frac{\mathrm{d}r_b}{\mathrm{d}t} = -\lambda r_b - f(r_u, r_b, a). \tag{7}$$

In Supplementary Equations (5-7) the terms -λX (with $X = a, r_b$ or r_u) describe the effective dilution due to growth and $s(\lambda) = \lambda r_{tot}$ is the ribosome synthesis rate. In balanced exponential growth, all time-derivatives in Supplementary Equations (7) vanish and the steady-state solution reads

$$\left(\frac{\lambda}{\lambda_0}\right)^3 - \left(\frac{\lambda}{\lambda_0}\right)^2 + \left(\frac{\lambda}{\lambda_0}\right)\left[\frac{1}{4}\left(\frac{\lambda_0^*}{\lambda_0}\right)^2 + \frac{a_{ex}}{2\text{IC}_{50}^*}\left(\frac{\lambda_0^*}{\lambda_0}\right)\right] - \frac{1}{4}\left(\frac{\lambda_0^*}{\lambda_0}\right)^2 = 0, \tag{8}$$

where $\lambda_0^* = 2\sqrt{p_{out}\kappa_t K_D}$ and $\text{IC}_{50}^* = \Delta r \lambda_0^*/(2p_{in})$. This equation can be recast into

$$c = \frac{1}{\alpha^2 + 1}\left(\frac{\alpha^2}{y} - \alpha^2 + 4y - 4y^2\right), \tag{9}$$

where

$$c = a_{ex}/\text{IC}_{50}, \tag{10}$$

$$y = \lambda/\lambda_0, \tag{11}$$

$$\alpha = \lambda_0^*/\lambda_0. \tag{12}$$

Here, IC_{50} is the concentration required to halve the growth rate (compared to no drug case) and we took into account that $\text{IC}_{50}/\text{IC}_{50}^* = (\alpha^2 + 1)/2\alpha$. Importantly, the dependence of the relative growth rate y on the relative concentration c dramatically changes when $\alpha < \alpha_{crit} = 2/3\sqrt{3} \approx 0.385$, as Supplementary Equation (9) exhibits a concentration interval in which growth rate has two stable solutions [2].

Pair of antibiotics When a pair of antibiotics is considered, additional ODEs are added to describe the binding of individual antibiotics to ribosomes (first binding step) as well as the simultaneous binding of two antibiotics

to the already bound ribosome (second binding step):

$$\frac{\mathrm{d}a_i}{\mathrm{d}t} = -\lambda a_i + f_i(r_\mathrm{u}, r_{\mathrm{b},i}, a_i) + \delta_{\mathrm{off},i} k_{\mathrm{off},i} r_\mathrm{b}^{a_1,a_2} - \delta_{\mathrm{on},i} k_{\mathrm{on},i} a_i r_{\mathrm{b},\bar{i}} + J_i(a_{\mathrm{ex},i}, a_i), \tag{13}$$

$$\frac{\mathrm{d}r_{\mathrm{b},i}}{\mathrm{d}t} = -\lambda r_{\mathrm{b},i} - f_i(r_\mathrm{u}, r_{\mathrm{b},i}, a_i) + \delta_{\mathrm{off},\bar{i}} k_{\mathrm{off},\bar{i}} r_\mathrm{b}^{a_1,a_2} - \delta_{\mathrm{on},\bar{i}} k_{\mathrm{on},\bar{i}} a_{\bar{i}} r_{\mathrm{b},i}, \tag{14}$$

$$\frac{\mathrm{d}r_\mathrm{b}^{a_1,a_2}}{\mathrm{d}t} = -\lambda r_\mathrm{b}^{a_1,a_2} + \sum_{i=A,B} \delta_{\mathrm{on},i} k_{\mathrm{on},i} a_i r_{\mathrm{b},\bar{i}} - \sum_{i=A,B} \delta_{\mathrm{off},i} k_{\mathrm{off},i} r_\mathrm{b}^{a_1,a_2} \tag{15}$$

$$\frac{\mathrm{d}r_\mathrm{u}}{\mathrm{d}t} = -\lambda r_\mathrm{u} + \sum_{i=A,B} f_i(r_\mathrm{u}, r_{\mathrm{b},i}, a_i) + s(\lambda). \tag{16}$$

In the system of Supplementary Equations (13-16), the kinetic parameters and the transport flux and binding functions depend on the antibiotic (indices i). The additional terms $\delta_{\mathrm{off},i} k_{\mathrm{off},i} r_\mathrm{b}^{a_1,a_2}$ and $\delta_{\mathrm{on},i} k_{\mathrm{on},i} a_i r_{\mathrm{b},\bar{i}}$ describe the rates of detachment of the i-th antibiotic from double-bound ribosomes $r_\mathrm{b}^{a_1,a_2}$ and binding of the i-th antibiotic to the ribosome already bound by the other antibiotic $\bar{i}$, respectively. The parameter $\delta_{j,i}$ determines the relative changes in rate constants when the other antibiotic is bound. Here, we investigated two cases: independent binding of the two antibiotics, i.e., $\delta_{j,i} = 1$ and competition $\delta_{j,i} = 0$, where binding of either antibiotic excludes binding of the other one. Setting the $\delta_{j,i} = 0$ results in additivity; the effects of different values of $\delta_{j,i}$ are presented in Ref. [2].

We obtained the steady-state solution of Supplementary Equations (13-16) numerically by forward time integration (Mathematica function NDSolve). While forward integration requires explicit values of kinetic constants k_on and K_D, the steady-state solutions are largely independent of the exact parameter values as long as the parameter ratios α and IC_{50}^* are the same and $k_\mathrm{on} \gg \kappa_\mathrm{t}$. Upon fitting α to the normalized dose-response curves, we fixed $k_\mathrm{on} = 100\ \mu\mathrm{M}^{-1}\mathrm{h}^{-1}$ (which gave consistent results for all dose-response curves). For each dose-response curve, we determined the optimized value of K_D—this was required due to the explicit need of parameters in forward integration (Supplementary Figure 2). By constraining these parameters, we can calculate the steady-state solutions of Supplementary Equations (13-16). We verified the impact of uncertainties in fitted response parameters by bootstrapping.

Ribosome subpopulations If we consider that translation inhibitors can bind only to a specific subpopulation of ribosomes, we can extend the model from above to incorporate this effect. In the following, we assume for simplicity that there are only two distinct subpopulations of ribosomes ($r_\mathrm{t,A}$ and $r_\mathrm{t,B}$) that can be bound by different antibiotics (A and B, respectively). Ribosomes cycle between two stages as per

$$\frac{\mathrm{d}r_\mathrm{t,A}}{\mathrm{d}t} = -k_\mathrm{A} r_\mathrm{t,A} + k_\mathrm{B} r_\mathrm{t,B}, \tag{17}$$

$$\frac{\mathrm{d}r_\mathrm{t,B}}{\mathrm{d}t} = k_\mathrm{A} r_\mathrm{t,A} - k_\mathrm{B} r_\mathrm{t,B}, \tag{18}$$

where k_A and k_B are the cycling rates. Here, assuming that cycling equilibrates quickly, the steady-state solution is simply $r_\mathrm{t,A} = \eta r_\mathrm{t,B}$, where $\eta = k_\mathrm{B}/k_\mathrm{A}$; because $r_\mathrm{u} - r_\mathrm{min} = r_\mathrm{t,A} + r_\mathrm{t,B}$ it follows that $r_\mathrm{t,B} = (r_\mathrm{u} - r_\mathrm{min})/(1+\eta)$ and $r_\mathrm{t,A} = (r_\mathrm{u} - r_\mathrm{min})\eta/(1+\eta)$. This effectively means that the binding rates are simply rescaled by these factors accordingly, namely $k'_\mathrm{on,A} = k_\mathrm{on,A}\eta/(1+\eta)$ and $k'_\mathrm{on,B} = k_\mathrm{on,B}/(1+\eta)$, where the primed constants are those that would be observed *in vivo*, whereas rates without a prime would be observed if a particular population of ribosomes in a specific stage is exposed to the antibiotic *in vitro*. As these considerations are valid for the individual antibiotics as well, these effects are already taken into account by fitting the response parameter α. Together with the assumption that the antibiotics bind only ribosomes in a specific stage, these considerations are equivalent to setting the $\delta_{\mathrm{on},i} = 0$, which results in additivity [2]. This example suggests that the possibility of partitioning ribosomes is already implicitly taken into account, yet it does not recover any secondary effect such as traffic jams, factor deprivations, etc. from which complex interactions might arise.

Clustering of bottleneck-dependency vectors

We performed the clustering of BD vectors projected on a space of lower dimensionality. For dimensionality reduction, we used Principal Component Analysis (PCA). We used the first three principal components which explained $\approx$ 95.38% of the variance. In this projected three-dimensional space, we performed unsupervised agglomerative clustering (Mathematica function FindClusters) with cosine distance as a measure of cluster cohesion.

We estimated the p-value of the observed clustering by bootstrapping. We used the Rand index (RI) [4] as a criterion for evaluating the difference between clustering results. For example, if w is the clustering obtained for the reshuffled sample and (consensus) clustering w' is obtained for PCA projection of median bottleneck dependency vectors (shown in Fig. 3 and Supplementary Figure 3), then the Rand index is

$$\mathrm{RI}(w, w') = \frac{\sum_{i<j}^{N} \psi_{ij}}{N(N-1)/2} \in [0, 1]. \tag{19}$$

Here, ψ_{ij} is 1 if the i-th and j-th data points are either inside or outside of the same cluster and 0 otherwise; the denominator is the total number of unique pairs between N elements. We generated 10^4 reshuffled datasets, evaluated RI for each clustering of the dataset, and calculated the cumulative distribution function. We evaluated an empirical p-value as

$$p = 1 - \mathrm{CDF}\left(1 - \frac{1}{N(N-1)/2}\right) \approx 3 \times 10^{-4}, \tag{20}$$

which is an estimate of the probability for obtaining the observed clustering of median BD vectors by chance. The cluster areas shown in Fig. 3 were obtained by smooth bootstrapping of median BD vectors for a given noise statistics, which were PCA projected, and subsequent calculation of the minimal convex hull (Mathematica function ConvexHullMesh). The additional response vectors for LAM, TMP, and NIT were PCA projected (using Mathematica function DimensionReduction obtained for the median values of BD vectors). Note, that the plots in Fig. 3e show projections onto PC1,2 but clustering was performed on the first three principal components (Supplementary Figure 3).

Remapping

Our remapping procedure converts inducer concentrations b into the concentrations c of an idealized antibiotic that precisely targets the translation step controlled by the titrated factor. This requires an induction curve and a dose-response curve. The former is described by an increasing Hill function $g(b)$, and the latter by solving Supplementary Equation (9) for y. The conversion between concentrations is formally described as $c = y^{-1}(g(b))$ at a given α, which can be arbitrarily chosen for the idealized antibiotic. When $\alpha < \alpha_{\mathrm{crit}}$, the dose-response curve is bistable and has a region in which more than one response will yield the same concentration—in these cases we consider only the concentration corresponding to the highest stable growth rate as the other solutions are either unstable or will be outcompeted. Further, higher inducer concentrations are remapped to lower antibiotic concentrations and an infinite inducer concentration corresponds to zero antibiotic concentration. As this is impractical, we considered all mimicked concentrations (normalized relative to IC_{50}) that are below 0.1 as equivalent to 0.

Regularization of surfaces

Strains containing the factor titration platform have mostly very similar antibiotic dose-response curves to the wild-type at maximal inducer concentrations. However, to correct for small deviations, we rescaled the antibiotic concentrations on the antibiotic-inducer grid. The shape of this transformation is derived from equating the responses of two Hill functions with different steepnesses. Consider two Hill functions with Hill exponents n_{WT} and n_{t}, for WT and factor-titrating strain, respectively. Then, by equating the responses captured by these Hill functions, we calculated the rescaled relative (relative to IC_{50}) antibiotic concentrations as $c_{\mathrm{a,WT}} = c_{\mathrm{a,t}}^{n_{\mathrm{t}}/n_{\mathrm{WT}}}$. We refer to this conversion as the "power-law transform". Such regularized surface was then used in remapping.

Remapping-based equivalence

Factor deprivation is equivalent to the action of a specific antibiotic if both perturbations can be substituted for each other. Upon remapping the inducer concentration, the response surface for an equivalent inducer-antibiotic pair is transformed into an additive response surface. To determine if the deprivation of a specific factor is equivalent to the action of a specific antibiotic, we performed the remapping in tandem with bootstrapping. Bootstrapping assesses the effects of uncertainties in the remapping parameter α (obtained as a response parameter α from a fit of inverted Supplementary Equation (9) to a drug dose-response curve), artifacts of the response surface over inducer-antibiotic grid and sampling, and inherent noisiness of growth rate determination. We first restricted the dataset to data points with relative growth equal to 0 or above 0.1 with growth rate coefficient of determination $R^2 > 0.8$. In each round of bootstrapping, the following steps are carried out:

1. drawing of a remapping parameter α from a normal distribution, centered at the best-fit-value and with standard deviation estimated from fitting, and remapping,
2. drawing of a random sample from remapped data points that is of random size (between 75% and 100% of the data set),
3. the addition of Gaussian noise to the growth rates (estimated from the growth rate fit),
4. calculation of the ideal additive surface at a given α for comparison, and
5. calculation of LI score.

This procedure was repeated 100 times for each bottleneck-antibiotic pair and yielded a set of distributions. Each LI distribution was then statistically evaluated for being inside the additive interval. We obtained the cumulative distribution function (CDF) for each distribution and we calculated its value on both ends of additive interval (Supplementary Figure 1). If either $1 - \mathrm{CDF}\left(b_{\mathrm{lower}}\right)$ or $\mathrm{CDF}\left(b_{\mathrm{upper}}\right)$ is below $p = 0.05$, the pair is considered inequivalent—this is the case in which the remapped surface is unlikely to be additive. For each antibiotic, more than one of the bottlenecks could be statistically equivalent—we thus deemed the bottleneck-antibiotic pair with the highest correlation between average remapped and ideal additive growth rates to be the primary candidate for equivalence of perturbations.

Quantitative comparison of predicted and measured response surfaces

Both measured and predicted surfaces match along the individual concentration axes, as these were obtained from the fits of dose-response curves. Thus, points corresponding to such measurements are always a good match and in turn increase the Pearson correlation invariantly of a potential mismatch in surface segments further away from individual axes. The Loewe interaction score is a good measure of interaction strength; however, it is not well suited for comparing the predicted and measured surfaces. Crucially, the same value of LI can be obtained for two very different surfaces. For example, if the same suppressive interaction surface is rotated by 90°, the LI score would remain the same, even though the direction of suppression is completely different. We therefore sought an applicable metric that would identify systematic deviations from predicted isoboles.

We developed an "isobole sliding" method in which we determine a mean deviation of points close to some predicted growth rate from measured values. It provides a concise, quantitative description of differences between predicted and measured isoboles and identifies the most discrepant areas of the surfaces. For this, we systematically move along the (ordered) predicted growth values g_i and select $S = 20$ consecutive points and average their deviations from measured values of growth rate h_i. This yields a deviation trajectory $t(\hat{g})$ of a mean deviation as a function of average predicted growth rate

$$t(\hat{g}) = \frac{1}{S}\sum_{i=j}^{j+S-1} (h_i - g_i), \quad \text{where} \quad g_i < g_{i+1} \ \text{and} \ \hat{g} = \frac{1}{S}\sum_{i=j}^{j+S-1} g_i. \tag{21}$$

Keeping the number of points S fixed in the window allows the comparison between different subsets of the data.

To assess the probability of observing such deviation by chance, we created a benchmark dataset by replacing all measured values with predicted ones to which we added Gaussian noise (estimated from bootstrapped dispersion, but of at least 0.05 relative growth units). For each bootstrapped realization (obtained either by remapping or the biophysical model), we randomly drew a subset of random size (between 75% and 100% of the data set) to estimate the robustness of the prediction concerning a low number of outliers. We collapsed each isobole sliding trajectory into a single number s by calculating a maximal deviation, $s = \max_{\hat{g}} |t(\hat{g})|$, thus yielding a distribution of s values for both measured and benchmark trajectory maxima.

Ideally, the distribution of maximal average deviations should either overlap or be below the benchmark distribution. To assess the statistical deviation, we evaluated the CDF of predicted-measured distribution at the 95% percentile of the benchmark distribution. If the value was below 0.05, we rejected the prediction. This method requires that there are no systematic deviations over the whole surface, thus yielding a very stringent criterion for considering a match between two surfaces. Thus, even if two surfaces match qualitatively, isobole sliding might still return a statistically significant mismatch.

To estimate the upper bound of prediction-measurement agreement, we checked for consistency of the measured replicates. For this, we considered one of the replicates as a prediction of the other. In doing so, we observed that twenty-one out of twenty-eight (75%) surfaces act as statistically significant predictions for one another. This serves as an approximate upper bound for how many predictions-measured pairs can be expected to match at the given experimental variability.

Assessment of predictive power

At this point, we can assess the consistency of predictions. Using the method described above, we evaluated both independent and competitive binding schemes for their congruence with measured surfaces. The scheme that led to the distribution with the smallest mean maximal deviation, was considered as a best-match. However, both schemes can yield a good match—by asking how many of the schemes yield a match in both replicates, we obtain an estimate for a fraction of correct predictions (Supplementary Figure 2). By counting cases in which at least one of the schemes yields a match between replicates, we find that sixteen out of twenty-eight interactions can be accounted for by a biophysical model.

Applying isobole sliding to the prediction of remapping shows that even small quantitative deviations will lead to the discarding of the prediction (Supplementary Figure 5). However, counting additionally explained interactions by remapping (TET-CRY, TET-FUS, KSG-CHL, CRY-KSG) increases the total tally of explained interactions to twenty out of twenty-eight (≈71.4%), which is below the estimated self-consistency bound of 75%. As discussed above, qualitative matches are not included in this metric.

TASEP model of translation within the growth law framework

There are several specific differences between the classical open TASEP system and translation in the context of the bacterial cell. Firstly, the pool of ribosomes is finite and variable in size (as dictated by the growth laws). Secondly, the ribosomes span over more than one site—it occupies 25 nucleotides, i.e., $L \approx 8.33$ codons [5, 6]. Thirdly, steps in the translation are mediated by translation factors that bind to the ribosome in a specific state and (stochastically) push the ribosome into another state. The rates depend on the abundance of ribosomes in a specific state and the abundance of the factor catalyzing the step. Thus, the rates, which are kept fixed in the classical TASEP, become variable and system-state dependent.

Mathematical framework

Analytical results for TASEP of extended particles In the absence of ribosome pausing, established analytical results for the TASEP of extended particles can be used [7, 8, 9, 10]. If the release of ribosomes at the

end of the transcript is not limiting, two different regimes of ribosome traffic exist, namely the initiation- and translocation-limited regime. These regimes are separated by a non-equilibrium phase transition. The current of ribosomes J in the two regimes is given by:

$$J_{\text{init}}(\zeta,\gamma) = \frac{\zeta\,(\gamma-\zeta)}{[\gamma+\zeta\,(L-1)]} \quad \text{and} \quad J_{\text{tran}}(\zeta,\gamma) = \frac{\gamma}{\left(1+\sqrt{L}\right)^2}, \tag{22}$$

where ζ and γ are initiation and translocation attempt-rates, respectively. Translocation and initiation-limited regime are separated by a continuous phase transition at $\zeta_{\text{crit}} = \gamma/(1+L^{1/2})$. The ribosome coverage density ρ reads:

$$\rho_{\text{init}}(\zeta,\gamma) = \frac{L\zeta}{[\gamma+\zeta\,(L-1)]} \quad \text{and} \quad \rho_{\text{tran}}(\zeta,\gamma) = \rho_{\max} = \frac{1}{1+1/\sqrt{L}}. \tag{23}$$

The elongation velocity (rate) u depends both on the current and the ribosome density $\rho_r = \rho/L$ via $u = Jk/\rho_r$, where k is the step size (1 aa or 1 codon). This in turn yields

$$u_{\text{init}}(\zeta,\gamma) = k(\gamma-\zeta) \quad \text{and} \quad u_{\text{tran}}(\zeta,\gamma) = k\frac{\gamma}{1+1/\sqrt{L}}. \tag{24}$$

Distribution of ribosomes across different classes The total ribosome concentration r_{tot} is

$$r_{\text{tot}} = r_i + r_{\text{tr}} + r_{\min}, \tag{25}$$

where r_i and r_{tr} are the concentrations of non-initiated and translating ribosomes, respectively. Translating ribosomes are distributed across numerous mRNA transcripts in the cell and their concentration can be written as:

$$r_{\text{tr}} = \frac{1}{V}\sum_{p}^{M}\rho_{r,p}D_p = \frac{1}{V}\sum_{p}^{M}\frac{\rho_p}{L}D_p \approx \frac{1}{V}M\frac{\rho}{L}\bar{D} = \Xi\rho_r\bar{D}, \tag{26}$$

where D_p and $\rho_{r,p}$ are the length and ribosome density on the p–th transcript, respectively, M is the total number of transcripts and V the cell volume ($\Xi = M/V$ is the concentration of transcripts). The density of ribosomes $\rho_r = \rho/L$ is a TASEP-derived quantity and depends on the initiation attempt rate ζ and translocation attempt rate γ. In the last step, we assumed for simplicity that the density of ribosomes across the transcripts does not vary significantly between transcripts. However, if transcripts differ in their ribosomes densities, those with higher densities will enter the translocation limiting regime (in which traffic jams form) already at a smaller decrease in translocation attempt rate. If those transcripts code for essential genes, this will correspondingly lead to a decrease in growth rate already at such smaller decreases in translocation attempt rate. Such traffic jams would still be relieved by lowering the initiation rate even though traffic jams have not developed on all other transcripts. Thus, the qualitative conclusions of the analysis below would still hold, but the results would be quantitatively different. However, taking differences between transcripts into account would require explicit modeling of individual transcripts and is beyond the scope of this work. Assuming similar ribosomes densities allows replacement of the sum with $M\bar{D}$, where $\bar{D}$ is the average length of transcripts being translated; the proteome-weighted average length is $\bar{D} \approx 209$ aa [11].

The growth rate is proportional to the elongation velocity of ribosomes along the transcript $u(\zeta,\gamma)$ and to the number of translating ribosomes. However, there is a limit for the maximal elongation rate $u_{\max}$ because other processes (e.g., charged tRNA delivery) become limiting at some point in a given nutrient environment. We estimated the maximal elongation rate from the Michaelis-Menten-like relation between RNA/protein (R/P) and translation rate obtained in Ref. [12]: $u = k_{\text{el}}(R/P)/[(R/P)+K_{\text{el}}]$, where $k_{\text{el}} = 22$ aa s^{-1} and $K_{\text{el}} = 0.11$. We calculated the theoretical $(R/P) = (R/P)_{\min} + \lambda_0/\kappa_t^{R/P} \approx 0.53$, where $\kappa_t^{R/P} = 4.5$ h^{-1} and $(R/P)_{\min} = 0.09$ [3]. Plugging this (R/P) into the Michaelis-Menten function for the translation rate, we obtain $u_{\max} \approx 18$ aa s^{-1}. Thus, the growth rate is given as

$$\lambda = \kappa_t r_{\text{tr}} \min\left[\frac{u(\zeta,\gamma)}{u_{\max}}, 1\right]. \tag{27}$$

However, the growth rate feeds back into the total ribosome concentration via the growth law as

$$r_{\mathrm{tot}} = r_{\mathrm{i}} + r_{\mathrm{tr}} + r_{\mathrm{min}} = r_{\mathrm{max}} - \lambda\Delta r\left(\frac{1}{\lambda_0} - \frac{1}{\kappa_{\mathrm{t}}\Delta r}\right). \tag{28}$$

We can estimate Ξ at λ_0 as

$$\frac{\lambda_0}{\kappa_{\mathrm{t}}} = \Xi\rho_{\mathrm{r}}\bar{D} \implies \Xi = \frac{\lambda_0}{\kappa_{\mathrm{t}}\rho_{\mathrm{r}}\bar{D}}. \tag{29}$$

Factor-dependent translocation attempt rate The ribosome will perform a specific step only when the associated factor is bound to it: the step-attempt rate is proportional to the probability P_{b} of the ribosome being bound by a factor, i.e., $\gamma = \gamma_0 P_{\mathrm{b}}$. This probability can be calculated by assuming a population of elongation factors with concentration $c_{\mathrm{ef}} = c_{\mathrm{ef,b}} + c_{\mathrm{ef,n}}$ and translating ribosomes $r_{\mathrm{tr}} = r_{\mathrm{tr,b}} + r_{\mathrm{tr,n}}$, where the indices b and n denote the factor-bound and unbound subpopulations, respectively. Binding is described by

$$\frac{\mathrm{d}r_{\mathrm{tr,b}}}{\mathrm{d}t} = k_{\mathrm{on}}c_{\mathrm{ef,n}}r_{\mathrm{tr,n}} - k_{\mathrm{off}}r_{\mathrm{tr,b}}, \tag{30}$$

$$\frac{\mathrm{d}c_{\mathrm{ef,b}}}{\mathrm{d}t} = k_{\mathrm{on}}c_{\mathrm{ef,n}}r_{\mathrm{tr,n}} - k_{\mathrm{off}}c_{\mathrm{ef,b}}. \tag{31}$$

Solving for the steady state, noting that $r_{\mathrm{tr,b}} = c_{\mathrm{ef,b}}$ and defining $K_{\mathrm{D}} = k_{\mathrm{off}}/k_{\mathrm{on}}$ we obtain the probability for a ribosome to be bound as

$$P_{\mathrm{b}} = \frac{r_{\mathrm{tr}} - r_{\mathrm{tr,n}}}{r_{\mathrm{tr}}} = 1 - \frac{(r_{\mathrm{tr}} - K_{\mathrm{D}} - c_{\mathrm{ef}}) + \sqrt{4K_{\mathrm{D}}r_{\mathrm{tr}} + (r_{\mathrm{tr}} - K_{\mathrm{D}} - c_{\mathrm{ef}})^2}}{2r_{\mathrm{tr}}}. \tag{32}$$

The binding constant of EF-G to the ribosome complex I (pre-translocation analog with N-Ac-dipeptidyl-tRNA at the A-site and deacylated-tRNA in the P-site) [13] is $K_{\mathrm{D}} = 0.27 \pm 0.02\ \mu$M; we used this value in our calculations.

Factor-dependent initiation attempt rate Successful initiation events are not limited to a single L-codon long slot on a mRNA (that can be free or occupied) but can occur on any transcript; and only the factor-bound ribosomes can attempt an initiation event. Thus, the initiation rate can be described by Michaelis-Menten kinetics:

$$\zeta = \zeta_0[\mathrm{IF}]\frac{r_{\mathrm{i}}}{K_{\mathrm{m}} + r_{\mathrm{i}}}. \tag{33}$$

We can estimate K_{m} from kinetic rates determined by Milon *et al* [14] where the free 30S subunit is bound (almost simultaneously) by IF3 and IF2 with the rate $(2-10)\times 10^2\ \mu\mathrm{M}^{-1}\mathrm{s}^{-1}$ and dissociates at the rate 30 s^{-1}. From these values, we estimate $K_{\mathrm{m}} \approx 0.05\ \mu$M.

Estimation of model parameters It is useful to estimate whether WT translation is in the initiation or translocation limited regime, which we can obtain from the average WT ribosome density. We can estimate the WT ribosome density as $\rho_{\mathrm{r}} = 3\beta_{\mathrm{r}}N_{\mathrm{r}}/(r_{\mathrm{m}}t_{\mathrm{m}})$, where N_{r}, β_{r}, r_{m}, and t_{m} are the number of ribosomes, the fraction of active ribosomes, the rate of mRNA synthesis per cell, and the average mRNA lifetime, respectively [15]. The fraction of translating ribosomes β_{r} is estimated from fitting a Hill function to data from Ref. [12] (Supplementary Figure 6).The average lifetime of mRNA is assumed to be growth rate-independent [16] at $t_{\mathrm{m}} \approx 1.5$ min [17]. For higher growth rates, the relationship between growth rate and (calculated) WT ribosome density linearizes; extrapolating to $\lambda_0 = 2.0\ \mathrm{h}^{-1}$, we obtain a rounded-up value of $\rho_{\mathrm{r}} \approx 0.07$ (Supplementary Figure 6), which yields $\Xi \approx 2.2\ \mu$M. For cells grown in LB, the average number of transcripts per cell was measured as $N_{\mathrm{mRNA}} \approx 7800$ [18]. To estimate the mRNA concentration, we use $\Xi = N_{\mathrm{mRNA}}/V_{\mathrm{cell}} = (N_{\mathrm{mRNA}}/m_{\mathrm{dry}}) \times (m_{\mathrm{dry}}/m_{\mathrm{wet}}) \times (m_{\mathrm{wet}}/V_{\mathrm{cell}})$, where $m_{\mathrm{dry}}/m_{\mathrm{wet}} \approx 1/3.1$ and $m_{\mathrm{wet}}/V_{\mathrm{cell}} \approx 1.09\ \mathrm{g\ mL}^{-1}$ are growth-rate independent quantities (see Supplementary Information of Ref. [1]). We obtained the dry mass of the cell at $\lambda = 2.0\ \mathrm{h}^{-1}$ by extrapolating from measured data at various growth rates [15] as $m_{\mathrm{dry}} \approx 1.01$ pg per cell (Supplementary Figure 6), which in turn yields $\Xi \approx 4.5\ \mu$M. This value is higher than our estimate, since the latter is skewed towards higher WT ribosome densities, which require fewer transcripts at the same number of translating ribosomes.

The estimated WT ribosome density is $\rho_r \approx 0.07$, which is lower than the maximal attainable ribosome density of $\rho_{r,max} = \rho_{max}/L = 1/(L + \sqrt{L})|_{L=25/3} = 0.089$. Thus, translation in the WT is likely in the initiation-limited regime. Thus, the equations for ribosomal density and elongation velocity for the initiation limiting regime are used to estimate the apparent initiation and translocation attempt rates:

$$\rho_r(\zeta) = \frac{\zeta}{\gamma + \zeta(L-1)} \approx 0.07 \quad \text{and} \quad u = (\gamma - \zeta)s \approx 18 \text{ aa/s}. \tag{34}$$

The apparent rates are $\gamma \approx 21.0\ \text{s}^{-1}$ and $\zeta \approx 3.0\ \text{s}^{-1}$. In the case of WT regulation there are ~ 0.83 EF-G molecules per ribosome and the expression of the factor is coupled to the ribosome number (i.e., their ratio is constant) [12]. This allows us to estimate $\gamma_0 = \gamma/P_b$, where we note that $c_{ef,WT} \approx 43.0\ \mu$M (estimated from $0.83 \times 51.9\ \mu$M where the ribosome concentration is calculated from the growth law). Next, we estimate the number of translating ribosomes from Supplementary Equation (27) as 32.6 μM, which yields $P_b \approx 0.98$ [Supplementary Equation (32)] and finally $\gamma_0 \approx 21.5\ \text{s}^{-1}$. We further note that there are 0.3 IF2 molecules per ribosome [15], implying $[\text{IF}]_{WT} \approx 15.6\ \mu$M, from which we estimate $\zeta_0 \approx \zeta/[\text{IF}]_{WT} \approx 0.19\ \mu\text{M}^{-1}\text{s}^{-1}$. With these parameter values, our model is fully defined and the growth rate is calculated (Mathematica function NSolve) as its output based on the concentration of translation factors.

We further explored the effects of parameters on the model results. First, we verified the impact of WT ribosome density ρ_r (one that supports maximal growth rate at saturating factor concentrations) and systematically calculated the response surfaces for different values of ρ_r between 0.0117 and 0.0817 (Supplementary Figure 6). With decreasing WT ρ_r, the concentration of mRNA Ξ increases according to Supplementary Equation (29). When $\Xi \gg \Delta r L/\rho_{max}\bar{D}$, the traffic jams of ribosomes are not possible anymore as there are too many mRNAs that can carry more ribosomes than available. The critical WT ribosome density is $\rho_{r,crit} = \lambda_0/(\kappa_t \Delta r) \times \rho_{r,max}$ (Supplementary Figure 6).

Next, we systematically calculated the response surfaces for different values of ribosome footprint size L and WT ribosome density ρ_r. Here, we varied ribosome footprint size L since: (i) a small increase in L could be caused by the space required by the movement of the ribosomal subunits during different phases of progression along mRNA, and (ii) size estimates in the literature vary [5, 9, 19, 20]. We varied L between 5 and 20 codons. We calculated the phase diagram of BD scores (Supplementary Figure 6). By doing so, we checked the impact of physiological parameters on the outcome of the model, and verified the mathematical relations discussed above more generally.

Supplementary Discussion

Antibiotic fingerprinting

To challenge the predictive power of translation bottlenecks, we tested whether the mode of action of a partially characterized antibiotic can be inferred from its bottleneck dependency vector. We focused on lamotrigine (LAM), an anticonvulsant drug that was recently identified to inhibit maturation and, in turn, reduce the number of translating ribosomes, potentially by interfering with initiation factor 2 (IF2, encoded by *infB*) [21]. The bottleneck dependency vector of LAM was most similar to that of KSG (Fig. 3d,e). As for LAM, a reduction of translating ribosomes is a signature of the initiation inhibitor KSG [22]. Hence, this observation further corroborates that similar bottleneck dependency vectors for translation inhibitors indicate a similar mode of action. Bottleneck dependency vectors indicated an unexpected similarity in an effective mode of action between ERM and KSG (Fig. 3d,e), an initiation inhibitor. These data suggest that ERM, which cannot block the synthesis of some proteins above a certain length [23], may effectively act as an initiation inhibitor. These results show that bottleneck dependency vectors often confirm established similarities but can also provide complementary insights into effective drug mode of action.

We further tested how an antibiotic with a mode of action unrelated to translation interacts with translation bottlenecks. If drug interactions are primarily determined by their mode of action [24, 25], antibiotics interfering

with processes unrelated to translation should be affected similarly by all different translation bottlenecks as the net effects of translation bottlenecks are indistinguishable—all lead to cessation of protein synthesis. To test this idea, we chose the antibiotic trimethoprim (TMP), which inhibits folate synthesis by binding to dihydrofolate reductase and is not known to directly perturb translation [26]. Its bottleneck dependency vector indicates that all bottlenecks alleviated TMP's action to various degrees (Fig. 3d)—a characteristic that is incompatible with any of the clusters of translation inhibitors (Fig. 3e). Furthermore, TMP is known to predominantly interact antagonistically or suppressively with translation inhibitors [24, 27]. These results support the idea that the effects of specific translation bottlenecks are diverse for antibiotics targeting translation, but not for antibiotics with modes of action unrelated to translation.

Streptomycin stands out among translation inhibitors as its action is aggravated by all translation bottlenecks (Fig. 3d). This might be a consequence of its multiple modes of action; besides interfering with tRNA binding, it slightly lowers the translocation rate [28] and causes protein mistranslation, changes in membrane potential, and membrane permeabilization [29]. These phenomena can lead to unspecific downstream effects in the cell. Therefore, we probed whether such unspecific effects can explain its distinct response to translation bottlenecks by measuring the bottleneck dependency vector of the prodrug nitrofurantoin (NIT). Nitrofurantoin has complicated effects on the bacterial cell, including the formation of non-native disulfide bonds in protein structures [30], DNA damage, and oxidative stress [31]. The similar bottleneck dependency between STR and NIT (Fig. 3d) is likely due to unspecific effects common to both drugs, such as the production of dysfunctional proteins.

Additive interactions and remapping

Our remapping approach further explained nontrivial additive interactions. In particular, the additive interaction between CHL and TET is hard to rationalize: these antibiotics have completely different binding sites on the ribosome. However, CHL and TET interacted similarly with translation bottlenecks (Fig. 3e) and their interaction was faithfully captured by the remapping approach (Fig. 5f). This observation suggests that the action of CHL is largely equivalent to inhibiting tRNA delivery. As CHL binding interferes with an aminoacyl moiety of tRNA on the A-site [32, 33], this suggests that perturbation of tRNA dynamics might be at the heart of the drug interaction between TET and CHL. At a more detailed level, CHL was shown to cause context-specific translation arrest by interfering with tRNA delivery only at specific codons [34, 35]. Our approach cannot capture such more detailed molecular aspects of the antibiotic mode of action; instead it indicates that the global effect of CHL is largely equivalent to hindered delivery of all tRNAs. The recently suggested effect of TET on initiation [36] is not reflected in our analysis, indicating at least that the effect of TET is not equivalent to initiation inhibition (Supplementary Figure 4).

KSG and ERM constitute another antibiotic pair that interacted additively and was clustered together (Fig. 3e). Remapping correctly predicted additivity between KSG-ERM (Supplementary Figure 5a); however, unlike KSG, ERM does not directly inhibit initiation (Table 1). Yet, ERM's inability to inhibit translation when the nascent peptide chain of some proteins [23] is extended beyond a certain length may effectively lead to a global functional equivalence with KSG, suggesting a possible cause of co-clustering and additivity between ERM and KSG (Fig. 3e). While plausible, these possibilities require further experimental investigation, especially since ERM selectively blocks the egress of peptide chains and reshapes the proteome [23, 37].

Effect of mRNA growth-rate dependence

The concentration of mRNA could in principle be growth rate-dependent. However, direct dependence of mRNA on the growth rate is difficult to estimate from existing literature as total RNA is mostly composed of rRNA and tRNA [3, 12]; estimation of the mRNA fraction is thus prone to errors. However, if we assume proportionality between ribosome and mRNA concentration, a simplified form can be written down as $\Xi = \Xi_0 r_{\mathrm{tot}}/r_{\mathrm{tot},0}$, where Ξ_0 and $r_{\mathrm{tot},0} = r_{\mathrm{min}} + \lambda_0/\kappa_{\mathrm{t}}$ are the estimates of mRNA concentration from the previous section and total ribosome

concentration in the unperturbed case, respectively. Plugging this dependence into the model does not qualitatively change the suppressive interaction between inhibition of initiation and translocation (Supplementary Figure 6). In this scenario, the increasing number of mRNA transcripts partially alleviates the densification of ribosomes on transcripts. However, the overall increasing number of translating ribosomes sequesters the elongation factors—this effect is still alleviated by lowering the initiation rate and in turn the density of ribosomes.

Rescue mechanisms and inefficiency of a direct response to translocation inhibition

Bacteria have evolved rescue mechanisms for stalled ribosomes (tmRNA, ArfA and ArfB). However, these mechanisms are mostly aimed at the rescue of ribosomes that were stalled due to limiting supply of building blocks or those in non-stop complexes. The former is an unlikely scenario during translocation limitation; as the building blocks are under-consumed, non-stop complexes can form via the formation of damaged or truncated mRNA (e.g., via cleavage by RNases) or via collision-induced frame-shifts [38, 39]. However, the tmRNA pathway requires an empty A-site on the ribosome, which is occupied in the pre-translocation complex, thus hindering the rescue initiation. Likewise, the ArfA pathway is hindered by an occupied A-site—it requires release factor 2 to bind to the A-site of the ribosome to initiate premature release and recycling. ArfB on the other hand, can recover the lack of tmRNA and ArfA pathways only when heavily overexpressed [40] and is considered ineffective in the WT regime. In sum, established rescue mechanisms are unlikely to recover stuck ribosomes and we therefore omit these mechanisms from the analysis.

Additionally, the cell could have an initiation-inhibiting mechanism in place as a response to translocation inhibition. However, the observed responses of bacteria to translation inhibition show global derepression of the translation machinery by reducing the levels of ppGpp. Besides the upregulation of all translation components mentioned in the main text [41, 42, 43, 44], an additional effect of lower levels of ppGpp is a direct increase of initiation. The catalytic function of the initiation factor is lowered when ppGpp levels are high, and higher when ppGpp is reduced [45]. These arguments show that an alleviating response of translocation inhibition by either rescue mechanisms or by direct down-regulation of initiation is unlikely.

Supplementary Tables

Type	Chemical name	Catalog numbers	Supplier
Antibiotics	Kanamycin sulfate	K4000-5G	Sigma-Aldrich
	Tetracycline hydrate	268054-25G	Sigma-Aldrich
	Streptomycin sulfate	S6501-25G	Sigma-Aldrich
	Lincomycin hydrocloride	62143-1G/5G	Sigma-Aldrich
	Fusidic acid sodium salt	F0881-1G/5G	Sigma-Aldrich
	Capreomycin sulfate	C4142-1G	Sigma-Aldrich
	Kasugamycin hydrochloride	K4013-10G	Sigma-Aldrich
	Erythromycin hydrate	856193-25G	Sigma-Aldrich
	Chloramphenicol	C0378-25G	Sigma-Aldrich
	Lamotrigine	2652	AvaChem
	Trimethoprim	92131-5G	Sigma-Aldrich
	Spectinomycin Sulfate	PHR1441-1H	Fluka
	Ampicillin sodium salt	A9518-5G	Sigma-Aldrich
Inducers	IPTG	2316.4	Roth
	Anhydrotetracycline	631310	Takara
	Anhydrotetracycline hydrochloride	37919-100MG-R	Sigma-Aldrich
Media	LB	L3022	Sigma-Aldrich

Supplementary Table 1: **Chemicals used in this study.** Table contains chemical names and purpose categories, catalog codes, and vendor information.

Supplementary Figures

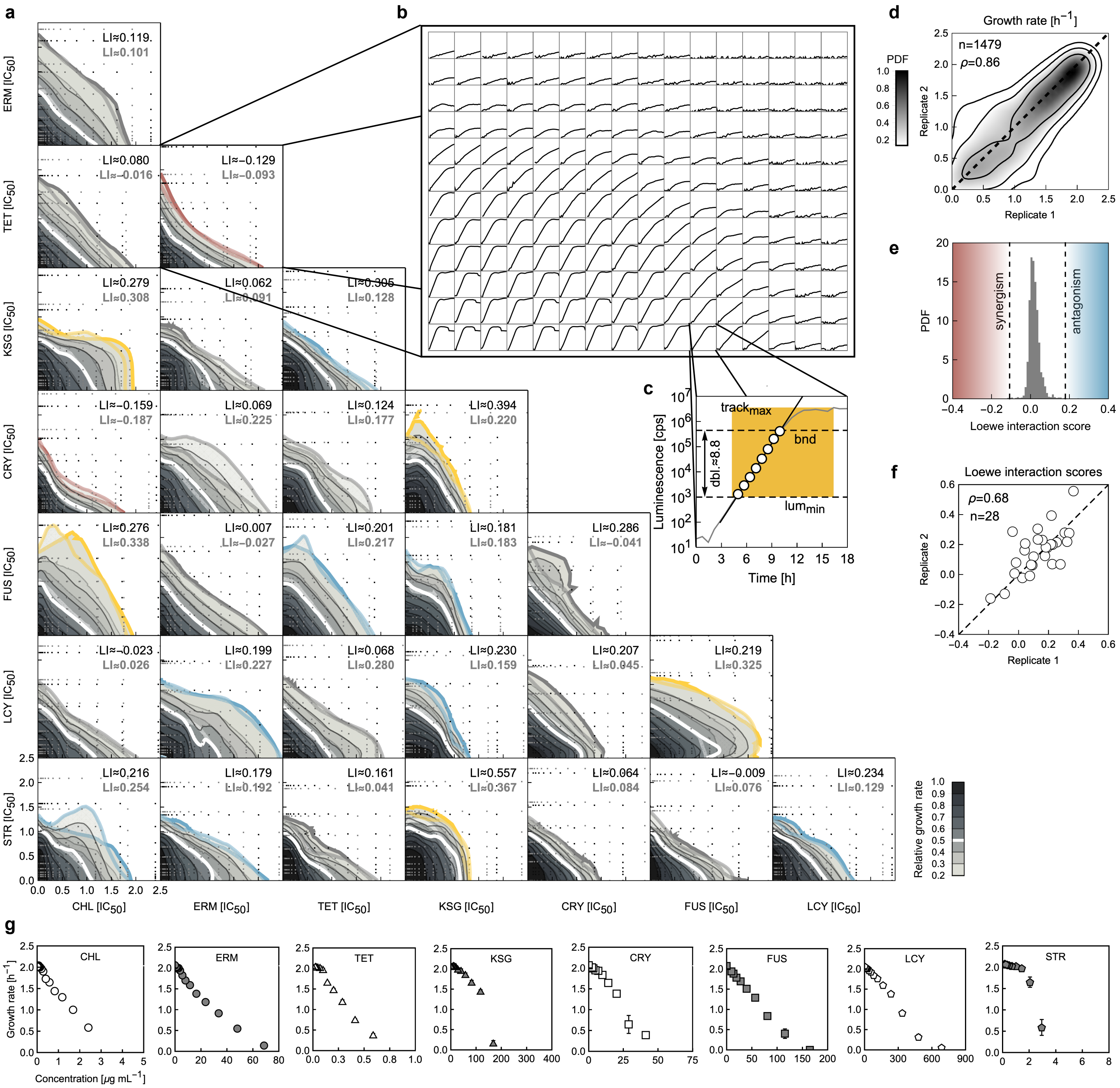

Supplementary Figure 1: **All dose-response surfaces and examples of growth curves.** See caption on the next page.

Supplementary Figure 1: **All dose-response surfaces and examples of growth curves.** **(a)** Duplicates of dose-response surfaces for all 28 antibiotic pairs. Due to small, but systematic variability in concentrations between replicates done on different days, we rescaled concentration axes relative to the IC_{50}. Dose-response surfaces were smoothed using LOESS (Methods). Black and gray dots denote measured points from different experiments. Isoboles from duplicates are in high agreement; small deviations are caused by occasional outliers that skew the isoboles. LI scores are shown for both replicates; gray score corresponds to the slightly transparent surface. **(b)** An example of growth curves over a 12×16 grid. Note, that here the concentrations geometrically change between wells , i.e., the ratio between concentrations in neighboring wells is fixed. **(c)** Exemplary growth curve and details of the fitting procedure. The growth rate is determined by fitting a line in the regime of exponential growth.The determination of this regime in the growth curve is carried out automatically; procedure: (i) check if the maximum value of luminescence is above the lower bound of the fitting interval $\text{lum}_{\text{min}} = 10^3$ cps and take points before the maximum, (ii) take points that are the latest to rise over lum_{min}, (iii) determine the upper limit (bnd) of the fitting interval to be either ten-fold above the lum_{min} (guaranteeing $\log_2 10 \approx 3.3$ doublings of a fitting interval) or eight-times less than the track maximum (three doublings away from saturation) and (iv) fit a line to the log-transformed values of the luminescence signal if there are at least three data points. If lum_{min} is not exceeded, the well is counted as having no growth; if any of the other criteria are not fulfilled, growth is characterized as undetermined. For strains with titratable factors, we slightly widened the fitting interval by decreasing the lum_{min} to 500 cps, and the number of doublings from saturation to ≈ 2.6. **(d)** Reproducibility of absolute growth rate measurements between replicates. The smooth kernel representation of replicate measurements (Mathematica function SmoothKernelDistribution), performed on different days and different plate arrangements, demonstrates a good agreement overall. Only non-zero growth rates of sufficient quality ($R^2 > 0.5$ and relative error < 0.5) are included, resulting in $n = 1479$ replicated growth rate measurements. **(e)** Distribution of Loewe interaction scores of noisy additive surfaces for pairs of drugs with different steepnesses, as obtained by bootstrapping. Note, that this reveals a slight bias towards antagonism. Here, LI scores were calculated for 2×10^3 bootstrap surfaces. See section "Loewe interaction score" for details. **(f)** Reproducibility of LI scores between replicates. Each LI score was evaluated for replicated measurements on different days for 28 different antibiotic combinations from (a). The indicated Pearson correlation can be interpreted as an upper bound for the correlation between any predicted and measured LI scores. **(g)** Dose-response curves for all antibiotics with absolute values of growth rate and drug concentration on the axes. Where error bars are invisible, they are smaller than the symbols. Each dose-response curve was measured $n = 7$ times and non-zero growth rates were used in the calculation of the mean and standard deviation shown in the plot.

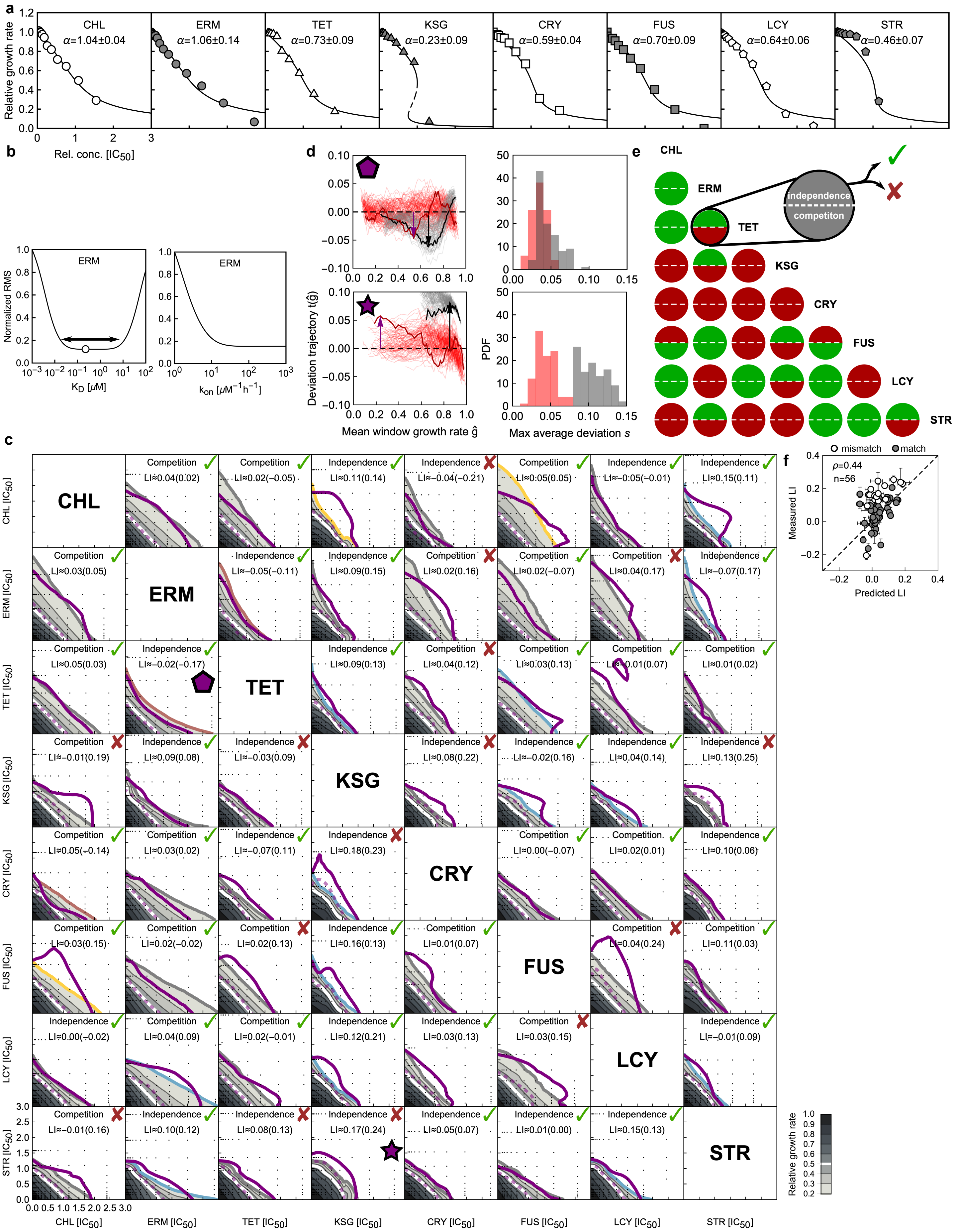

Supplementary Figure 2: **Details of the biophysical model for pairwise antibiotic combinations.** See caption on the next page.

Supplementary Figure 2: **Details of the biophysical model for pairwise antibiotic combinations.** **(a)** Average dose-response curves with the best fit model for individual antibiotics. Concentration axes of dose-response curves from Supplementary Figure 1g were rescaled by the respective IC_{50}. Insets denote the corresponding antibiotic and best-fit response parameter α with standard error. Dose-response curves are predominantly shallow for our selection of antibiotics, i.e., $\alpha > \alpha_{\text{crit}}$. The dashed segment of the KSG dose-response curve represents an unstable solution. **(b)** Example of an effect of numerical parameters (K_D and k_{on}) on root-mean-square error (in comparison to the experimental data). Parameters are required for forward time integration. The root-mean-square error was normalized relative to the maximal error in the scanned interval. Effective dissociation constant K_D exhibits roughly two orders of magnitude wide plateau (double-headed arrow; minimum is denoted by a circle). First-order binding rate constant k_{on} does not exhibit a plateau but rather flattens out—consistently with the requirement that $k_{on} \gg \kappa_t$. **(c)** All predictions for replicated measurements. Predicted surface is shown in full; overlaid thick and dashed purple isobole denote 20% and 50% isobole, respectively, of the measured surface. Each prediction is evaluated for goodness of prediction as described in Methods. Checkmark and cross denote a match and mismatch, respectively. Inset text denotes the best-matching binding scheme. The bootstrapped-LI scores for predicted and measured (in parentheses) surfaces are shown. **(d)** Illustration of isobole sliding method. Left: two examples of deviation trajectories $t(\hat{g})$ for ERM-TET (pentagram) and KSG-STR (five-point star). Thin gray and red lines show $n = 100$ bootstrap repetitions of predicted and benchmark trajectories. Two trajectories (thick black and red lines for measured and benchmark, respectively) are highlighted. Black and purple arrows denote the maximal deviation of the trajectory from zero for predicted and benchmark trajectory. The length of the arrow is maximal average deviation s. Right: all s values from $n = 100$ bootstrapped repetitions are collected in the histogram. The pair of ERM-TET offers a better match with benchmark distribution compared to KSG-STR. **(e)** Performance of all schemes against the measurements. The upper and bottom half of each circle denote independence or competition, respectively, as denoted. Green and red color denote match and mismatch, respectively. Match means that both replicates agree that a certain scheme faithfully predicts observed surface. **(f)** A scatter-plot shows median values of $n = 56$ predicted and measured LI scores for all possible predictions from (c). Error bars show 90% bootstrap confidence intervals obtained from 100 bootstrap repetitions. White and gray symbols correspond to match and mismatch cases, respectively; the LI score was computed for the best prediction with the binding scheme as in (c). Note, that this correlation is not expected to exceed that of replicated LI scores in Supplementary Figure 1f.

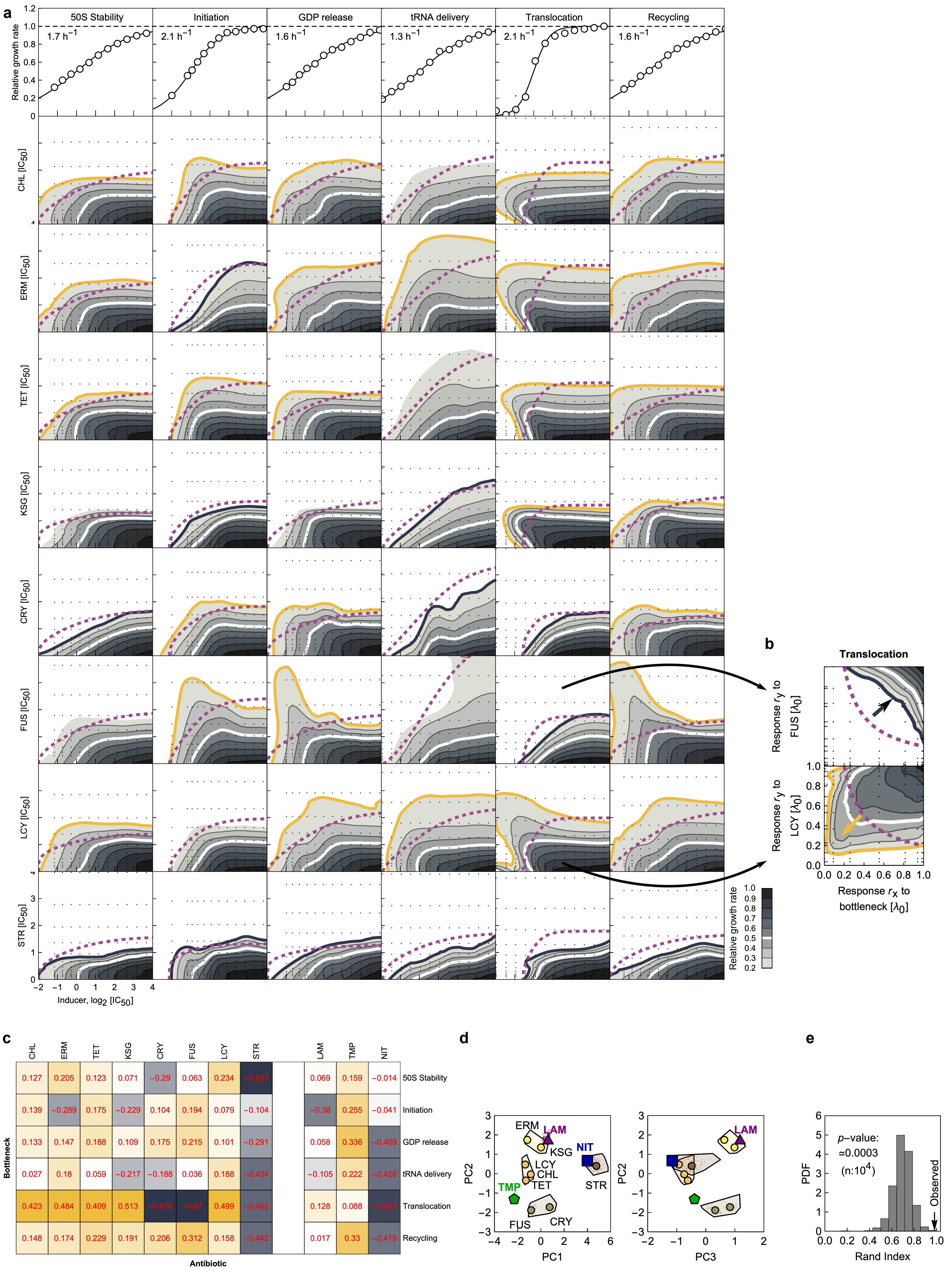

Supplementary Figure 3: **Bottleneck-antibiotic dose-response surfaces and functional classification.** See caption on the next page.

Supplementary Figure 3: **Bottleneck-antibiotic dose-response surfaces and functional classification.** **(a)** Dose-response surfaces for all bottleneck-antibiotic pairs. Surfaces were smoothed using LOESS (Methods). Note the different characters of deviations from independence. **(b)** Examples of response surfaces over the response-response grid. In the response space (r_x,r_y), independence is defined as $r_x r_y$. The logarithm of the ratio of volumes underneath the measured and independent surface yields a bottleneck dependency score. For every antibiotic, six bottleneck dependency scores together yield a bottleneck dependency vector. **(c)** Values of bottleneck dependency scores for all bottleneck-antibiotic pairs. **(d)** Projection of bottleneck dependency vectors on PCA vectors. Left: as in Fig. 3e. Right: projection on PCA vectors PC2 and 3. Note the separation of clusters in both projections. **(e)** Bootstrapped clustering of randomized vectors yields a series of clustering results. With these clustering results at hand, we calculate the Rand indices $\mathrm{RI}(w, w')$ for $n = 10^4$ bootstrap replicates. From the distribution of $\mathrm{RI}(w, w')$, we estimate the empirical cumulative distribution function and corresponding empirical p-value for the clustering result in Fig. 3e as $p = 1 - \mathrm{CDF}\,(1 - 1/[N(N-1)/2])$ [Supplementary Equation (20)]. This test is one-sided by construction as we ask how many clustering results have $\mathrm{RI} = 1$, which is the maximal attainable value.

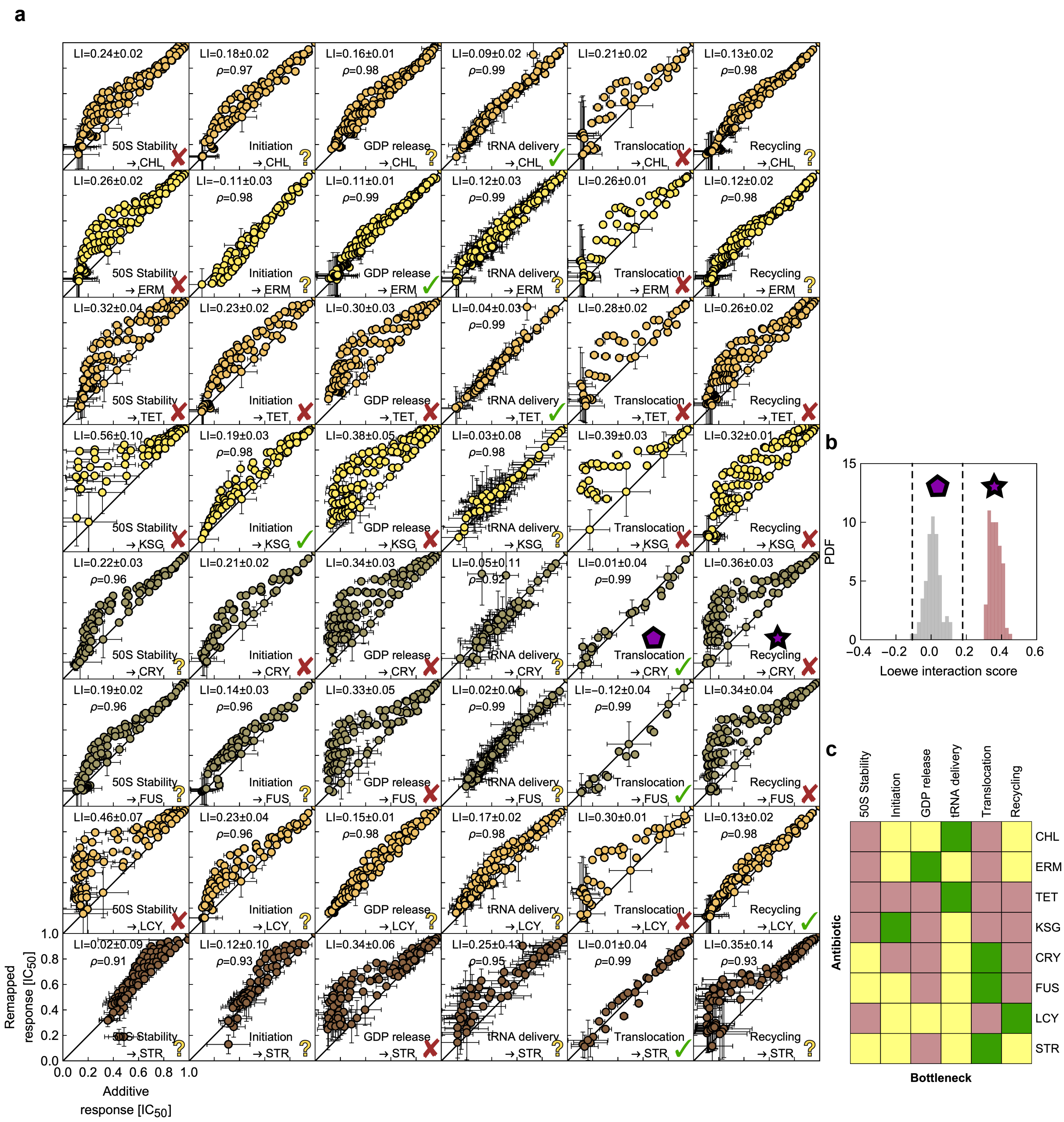

Supplementary Figure 4: **Remapping-based assessment of primary mode of action. (a)** Scatter plots of growth rates expected for additivity and obtained by self-remapping (Supplementary Methods). Error bars correspond to the standard deviation of $n = 100$ bootstrap repetitions. The distribution of LI was compared statistically to the boundaries of the additive interval. Red crosses denote that LI fell outside the additive interval. Green checkmarks and yellow question-marks denote that LI did not fall outside of the additivity interval; in these cases, the rounded correlation ρ is reported. For a given antibiotic, the bottleneck with the highest correlation with the additive expectation is marked with a checkmark and suggests the equivalency of antibiotic and genetic perturbation. **(b)** Examples of histograms of LI for CRY in combination with a translocation and recycling bottleneck [see matching pentagon and star in (a)], respectively. Each histogram is calculated for $n = 100$ bootstrapped LI scores. **(c)** Color-coded sequential evaluation of equivalence between bottleneck and translation inhibitor from (a). Red and yellow denote that LI was outside or inside of the additive interval, respectively. From the cases in which the LI is statistically inside the additive interval, the case with highest correlation was chosen as the putative primary mode of action (green). This approach correctly identified the mode of action for all cases in which it is known from the literature (CRY, FUS, STR, KSG, and TET).

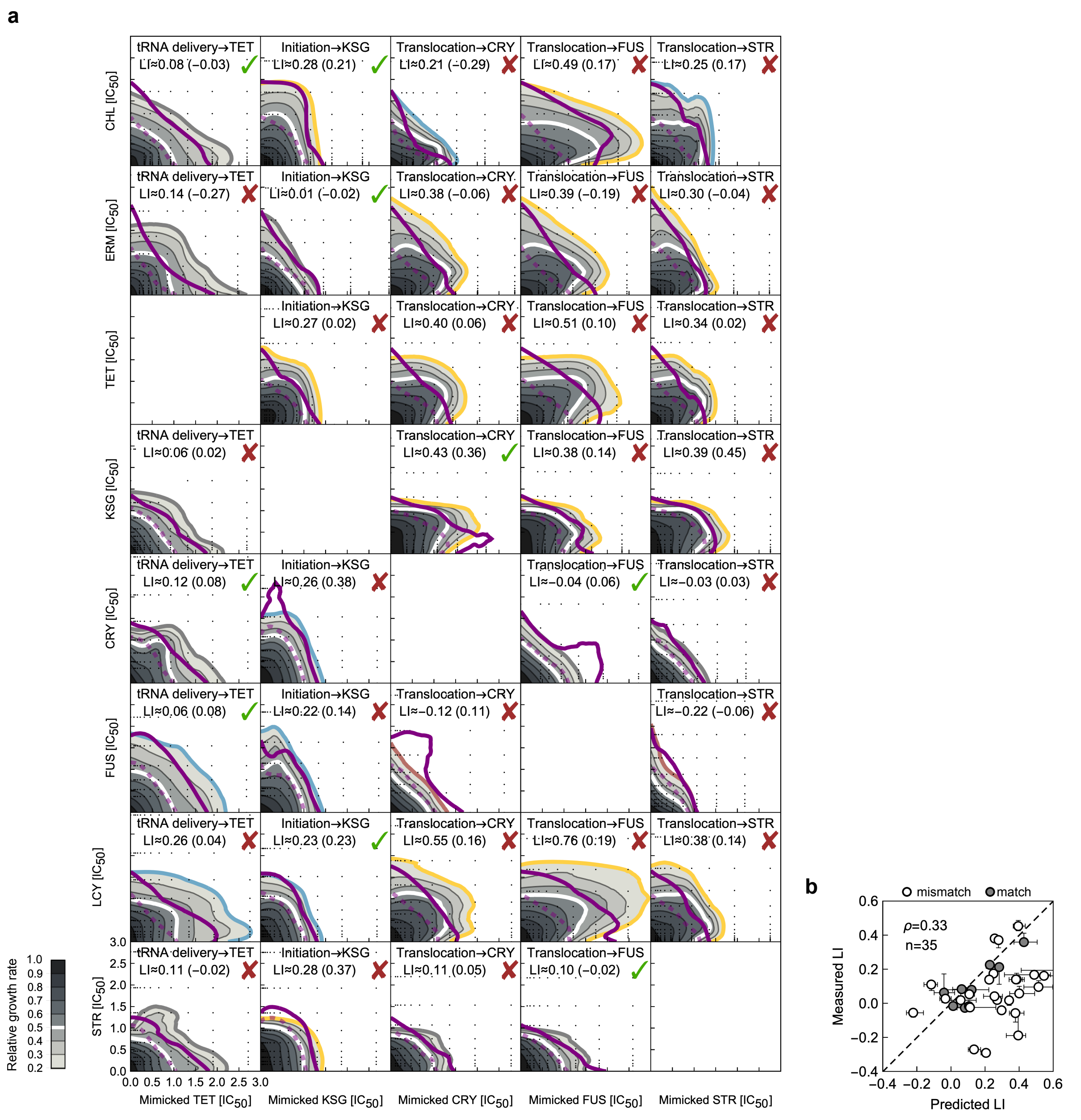

Supplementary Figure 5: **All possible predictions from perturbations of equivalent effects. (a)** The predicted surfaces obtained by remapping are shown in full; overlaid thick and dashed purple contour denote 20% and 50% isobole, respectively, of the measured surface. Each prediction is evaluated for the goodness of prediction as described in Supplementary Methods. Checkmark and cross denote a match and mismatch, respectively. **(b)** Scatter plot shows median values of $n = 35$ predicted and measured LI scores for all possible predictions from (a). Error bars denote 90% bootstrap confidence intervals. Medians and confidence intervals were estimated from 100 bootstrap repetitions. White and gray symbols correspond to match and mismatch cases, respectively, according to the criteria in Supplementary Figure 2d.

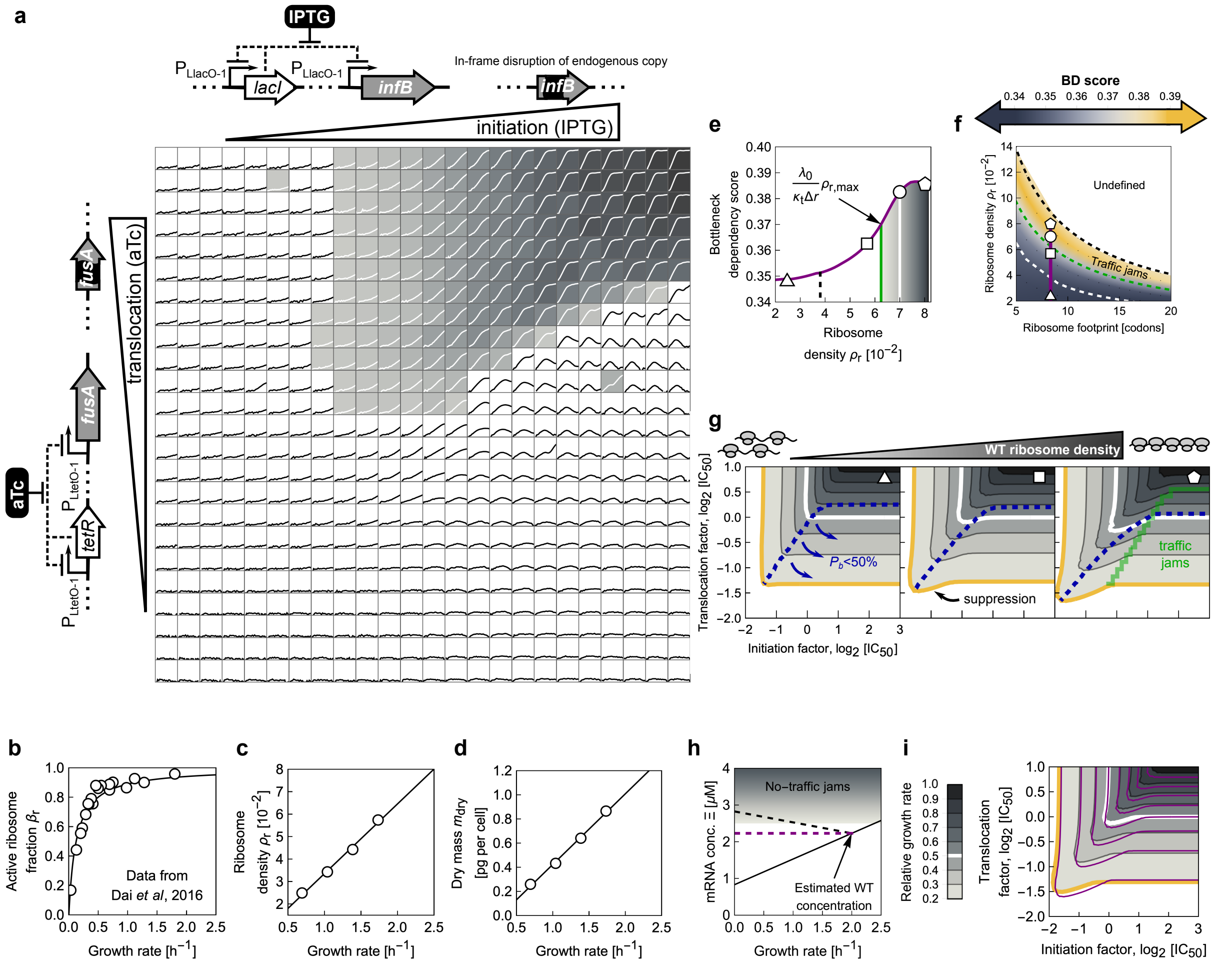

Supplementary Figure 6: **Double titration platform and model analysis.** See caption on the next page.

Supplementary Figure 6: **Double titration platform and model analysis.** **(a)** Schematics represent the genetic elements of double titration control: negatively auto-repressed transcription factors *lacI* and *tetR* that control the expression of initiation factor 2 (*infB*) and elongation factor G (*fusA*), respectively; expression is dependent on the shown inducers (IPTG and aTc). The grid shows the growth curves for the response surface in Fig. 6. Different shades of gray show the growth rate. Only fits of good quality and with growth rates above 0.199 are included. **(b)** Active ribosome fraction as a function of growth rate in different nutrient environments. Data is from Ref. [12]. The solid line represents a best-fit Hill function $(x/a)/[1+(x/a)]$, where $a \approx 0.12\ \mathrm{h}^{-1}$. **(c)** Calculated ribosome density $\rho_\mathrm{r} = 3\beta_\mathrm{r} N_\mathrm{r}/(r_\mathrm{m} t_\mathrm{m})$. The solid line shows best fit. **(d)** Dry mass measurements from Ref. [15] and best-fit linear function (solid line). Arrow denotes the density for $\lambda_0 = 2.0\ \mathrm{h}^{-1}$. **(e)** Impact of varying the initial ρ_r on resulting bottleneck dependency score for $L = 25/3$. White symbols correspond to the examples showcased in (f,g). The white circle shows the result from the main text for the estimated value of WT $\rho_\mathrm{r} = 0.07$. The solid green vertical line denotes the critical value $\lambda_0\rho_{\mathrm{r,max}}/(\kappa_\mathrm{t}\Delta r)$ above which traffic jams due to translocation limitation can form. The dashed vertical line indicates the approximate point at which suppression starts to develop due to factor sequestration (as obtained by $\partial\mathrm{BD}/\partial\rho_\mathrm{r} = 0.3$). **(f)** Phase diagram of BD score as a function of WT ribosome density ρ_r and ribosome footprint size L. Purple line and symbols corresponds to the cross-section in (e). The "undefined" area corresponds to the example in which translation is already in the regime with traffic jams and Supplementary Equations (34) do not have a unique solution. White dashed line corresponds to $\partial\mathrm{BD}/\partial\rho_\mathrm{r} = 0.3$, above which (approximately) suppression starts to develop. Green dashed line shows the $\lambda_0\rho_{\mathrm{r,max}}/(\kappa_\mathrm{t}\Delta r)$, above which traffic jams can develop. **(g)** Response surfaces for $\rho_\mathrm{r} \approx 0.025,\ 0.057,$ and 0.080 [from left to right; symbols shown as in (e,f)]. Symbols in the upper-right corner correspond to the values shown in (e,f). Dashed blue lines show where 50% of the translating ribosomes are bound by the elongation factor; below this line, this fraction decreases along the arrows. When ρ_r increases, suppression starts to develop. Increasing ρ_r further facilitates strong suppression by the formation of traffic jams (below green line). **(h)** Two models of mRNA concentration dependence. Black lines denote the dependence of mRNA on growth rate if the co-regulation between total RNA and mRNA (Supplementary Discussion) is assumed; solid and dashed lines correspond to variation of the nutrient quality and translation perturbation, respectively. The arrow denotes the estimated mRNA concentration (Supplementary Methods); this concentration is assumed constant (dashed purple line) in the model shown in the main text. If the mRNA concentration exceeds $\Delta r/\left(\rho_{\mathrm{max,r}}\bar{D}\right)$, traffic jams do not develop. Elongation factors are still sequestered as the number of translating ribosomes increases, which in turn decreases the growth rate. **(i)** Direct comparison of model predictions. Prediction with growth-dependent mRNA concentration Ξ is depicted in grayscale; isoboles from the prediction assuming a constant pool of mRNA are shown in purple. Both results are qualitatively equivalent.

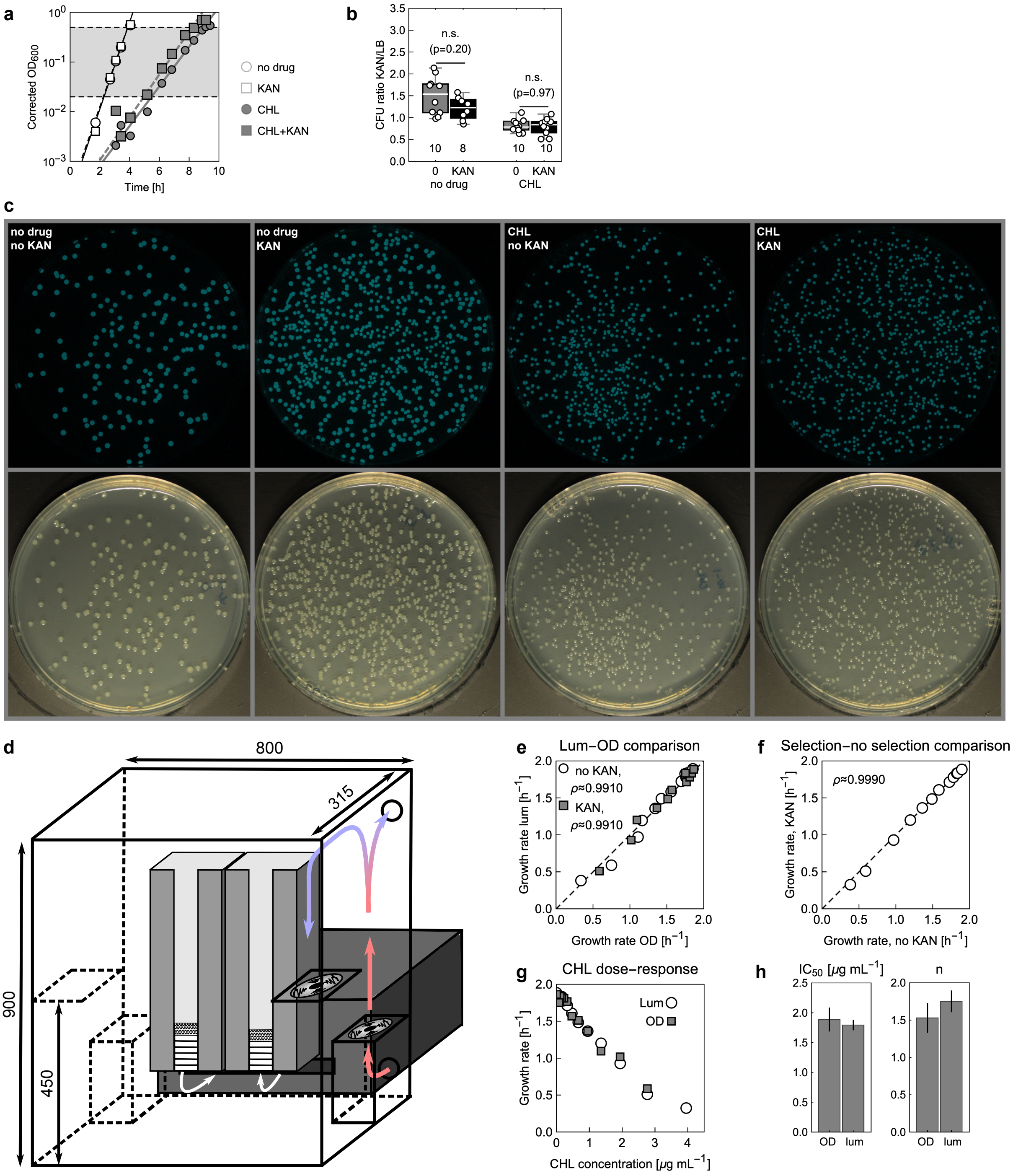

Supplementary Figure 7: **Verification of the growth-measurement technique and stacker-based setup.** See caption on the next page.

Supplementary Figure 7: **Verification of the growth-measurement technique and stacker-based setup.** **(a)** Time courses of optical density (OD) measurements of batch cultures in shaking flasks with or without selection for pCS-λ by kanamycin (KAN) and with or without antibiotic stress [2 μg mL^{-1} chloramphenicol (CHL)]. Selection does not alter the response to the CHL. Lines shown in the plot were fitted to log-transformed OD values from the shaded area. **(b)** The ratio of colony-forming units (CFUs) on selective (KAN) and non-selective (LB) agar plates obtained from batch cultures from (a). Cultures grown in the absence of selection do not significantly differ from those with applied selection, indicating that the loss of plasmid is non-significant (p-values obtained by the two-sided Mann-Whitney test, Mathematica function MannWhitneyTest). Box-and-whisker charts: whiskers extended to the full range of data points, box edges show a 25%-75% range, and centerline is a median value. The number under the boxes denote the number of independent technical replicates, whose values are overlaid on the boxes. **(c)** Examples of LB agar plates for four different conditions in (a), imaged with a camera in a bright-field mode with a long-exposure setting (30 s) to detect luminescence; all colonies are luminescent. **(d)** Stacker-based setup and the incubator box. Microtiter plates are stacked vertically in stacker towers and cycled (white arrows) through the Tecan M1000 plate reader (dark grey box) in which plates are shaken and the luminescence is measured. After all plates are stacked in the right tower, the stacking mechanism re-stacks the plates into the left tower to repeat the measurement. Fans circulate the air in the incubator to equilibrate the temperature. **(e)** Comparison of OD and luminescence-based growth rates for the CHL dose-response curve in the presence or absence of selection. Scatterplot reveals high-correlation between measurements, irrespective of selection. **(f)** Comparison of luminescence-based growth rates in the presence or absence of selection. **(g)** Dose-response curve for CHL, obtained by OD or luminescence assay. Qualitatively, curves match to a high degree. **(h)** Shape parameters of the dose-response curves as obtained from curves in (g). Height of bars correspond to the best-fit estimate for the curve parameter and black lines show standard errors as obtained from fitting. Number of measurements used in fitting were 11 and 12 for OD and luminescence assay, respectively, shown in (g).

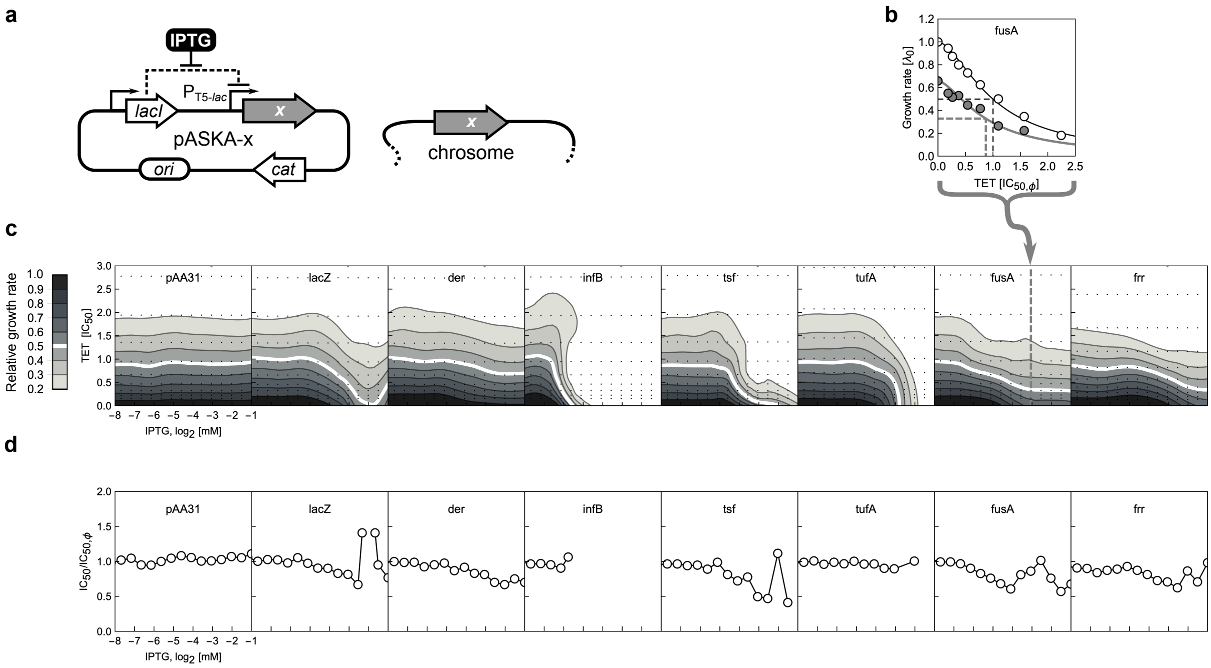

Supplementary Figure 8: **Overexpression of translation factors conveys little information about the action of tetracycline. (a)** The overexpression system is based on ASKA library plasmids. A high-copy plasmid with chloramphenicol resistance (cat) provides a constitutively expressed *lacI* repressor, which controls the expression of P_{T5-lac}-driven gene *x*. The chromosomal copies of the translation factors were left intact. The high copy number of the plasmid enables the overexpression of proteins. **(b)** Example of the tetracycline dose-response curve in the presence or absence of *fusA* overexpression. White and gray circles denote measurements of the dose-response curve in the absence and presence of inducer, respectively. Best-fit Hill functions are shown as solid lines. Dashed lines denote the apparent IC_{50} and corresponding growth rate compared to the no antibiotic at both inducer concentrations. The growth rate is decreased as a consequence of overexpression. Gray dose-response curve is a cross-section of the dose-response surface shown in (c): see the arrow. **(c)** Dose-response surfaces for gene-less control (pAA31), protein-overexpression control (*lacZ*) and all six translation factors as indicated. In general, the growth rate decreases with overexpression and the action of the antibiotic is not alleviated by overexpressing any of these genes. Cross-section of the dose-response surface shown in (b) for *fusA* is indicated by a dashed gray line. **(d)** Dependencies of the $IC_{50}/IC_{50,\phi}$ ratios ($IC_{50,\phi}$ corresponds to IC_{50} in the absence of inducer) for different overexpression cases as a function of inducer concentration. At each inducer concentration, the apparent IC_{50} is determined from a fit of a Hill function. In general, overexpression leads to mild sensitization of bacteria to tetracycline and this trend is largely independent of a specific overexpressed gene. The occasional slight increase is mostly due to the poor fits of the dose-response curve or the occurrence of spontaneous mutants.

References

[1] Greulich, P., Scott, M., Evans, M. & Allen, R. Growth-dependent bacterial susceptibility to ribosome-targeting antibiotics. *Mol. Syst. Biol.* **11**, 796–807 (2015).

[2] Kavčič, B., Tkačik, G. & Bollenbach, T. Minimal biophysical model of combined antibiotic action. `https://www.biorxiv.org/content/10.1101/2020.04.18.047886v2` (2020).

[3] Scott, M., Gunderson, C., Mateescu, E., Zhang, Z. & Hwa, T. Interdependence of cell growth and gene expression: Origins and consequences. *Science* **330**, 1099–1102 (2010).

[4] Rand, W. Objective criteria for the evaluation of clustering methods. *J. Am. Stat. Assoc.* **66**, 846–850 (1971).

[5] Mohammad, F., Green, R. & Buskirk, A. A systematically-revised ribosome profiling method for bacteria reveals pauses at single-codon resolution. *eLife* **8:e42591** (2019).

[6] Woolstenhulme, C., Guydosh, N., Green, R. & Buskirk, A. High-precision analysis of translational pausing by ribosome profiling in bacteria lacking EF. *Cell Rep.* **11**, 13–21 (2015).

[7] Klumpp, S. & Hwa, T. Stochasticity and traffic jams in the transcription of ribosomal rna: Intriguing role of termination and antitermination. *Proc. Nat. Acad. Sci. USA* **105**, 18159–18164 (2008).

[8] Lakatos, G. & Chou, T. Totally asymmetric exclusion processes with particles of arbitrary size. *J. Phys. A: Math. Gen.* **36**, 2027–2041 (2003).

[9] Shaw, L., Zia, R. & Lee, K. Totally asymmetric exclusion process with extended objects: A model for protein synthesis. *Phys. Rev. E* **68**, 021910 (2003).

[10] Zia, R., Dong, J. & Schmittmann, B. Modeling translation in protein synthesis with TASEP: A tutorial and recent developments. *J. Stat. Phys.* **144**, 405–428 (2011).

[11] Milo, R. & Phillips, R. *Cell Biology by the Numbers* (Garland Science, Taylor & Francis group; New York, 2016).

[12] Dai, X. *et al.* Reduction of translating ribosomes enables *Escherichia coli* to maintain elongation rates during slow growth. *Nat. Microbiol.* **2**, 16231 (2016).

[13] Yu, H., Chan, Y.-L. & Wool, I. The identification of the determinants of the cyclic, sequential binding of elongation factors Tu and G to the ribosome. *J. Mol. Biol.* **386**, 802–813 (2009).

[14] Milon, P. *et al.* Real-time assembly landscape of bacterial 30s translation initiation complex. *Nat. Struct. Mol. Biol.* **19**, 609–616 (2012).

[15] Bremer, H. & Dennis, P. Modulation of chemical composition and other parameters of the cell by growth rate. In Neidhardt, F. (ed.) *Escherichia coli and Salmonella* (ASM Press, Washington DC, 1996).

[16] Klumpp, S., Zhang, Z. & Hwa, T. Growth rate-dependent global effects on gene expression in bacteria. *Cell* **130**, 1366–1375 (2009).

[17] Yu, J., Xiao, J., Ren, X., Lao, K. & Xie, X. Probing gene expression in live cells, one protein molecule at a time. *Science* **311**, 1600–1603 (2006).

[18] Bartholomäus, A. *et al.* Bacteria differently regulate mRNA abundance to specifically respond to various stresses. *Phil. Trans. R. Soc.* **374**, 20150069 (2016).

[19] Kang, C. & Cantor, C. Structure of ribosome-bound messenger RNA as revealed by enzymatic accessibility studies. *J. Mol. Biol.* **181**, 241–251 (1985).

[20] Mitarai, N., Sneppen, K. & Pedersen, S. Ribosome collisions and translation efficiency: Optimization by codon usage and mRNA destabilization. *J. Mol. Biol.* **382**, 236–245 (2008).

[21] Stokes, J. *et al.* Discovery of a small molecule that inhibits bacterial ribosome biogenesis. *eLife* **3:e03574** (2014).

[22] Kaberdina, A., Szaflarski, W., Nierhaus, K. & Moll, I. An unexpected type of ribosomes induced by kasugamycin: A look into ancestral times of protein synthesis? *Mol. Cell* **33**, 227–236 (2009).

[23] Kannan, K., Vazquez-Laslop, N. & Mankin, A. Selective protein synthesis by ribosomes with a drug-obstructed exit tunnel. *Cell* **151**, 508–520 (2012).

[24] Yeh, P., Tschumi, A. & Kishony, R. Functional classification of drugs by properties of their pairwise interactions. *Nat. Genet.* **38**, 489–494 (2006).

[25] Brochado, A. *et al.* Species-specific activity of antibacterial drug combinations. *Nature* **559**, 259–263 (2018).

[26] Walsh, C. *Antibiotics: Actions, Origins, Resistance* (ASM Press, Washington DC, 2003).

[27] Bollenbach, T., Quan, S., Chait, R. & Kishony, R. Nonoptimal microbial response to antibiotics underlies suppressive drug interactions. *Cell* **139**, 707–718 (2009).

[28] Peske, F., Savelsbergh, A., Katunin, V., Rodnina, M. & Wintermeyer, W. Conformational changes of the small ribosomal subunit during elongation factor G-dependent tRNA–mRNA translocation. *J. Mol. Biol.* **343**, 1183–1194 (2004).

[29] Davis, B. Mechanism of bactericidal action of aminoglycosides. *Microbiol. Rev.* **51**, 341–350 (1987).

[30] Bandow, J., Brätz, H., Leichert, L., Labischinski, H. & Hecker, M. Proteomic approach to understanding antibiotic action. *Antimicrob. Agents Chemother.* **47**, 948–955 (2003).

[31] Mitosch, K., Rieckh, G. & Bollenbach, T. Noisy response to antibiotic stress predicts subsequent single-cell survival in an acidic environment. *Cell Syst.* **4**, 1–11 (2017).

[32] Wilson, D. Ribosome-targeting antibiotics and mechanisms of bacterial resistance. *Nature Rev. Microbiol.* **12**, 35–48 (2014).

[33] Dunkle, J., Xiong, L., Mankin, A. & Cate, J. Structures of the *Escherichia coli* ribosome with antibiotics bound near the peptidyl transferase center explain spectra of drug action. *Proc. Nat. Acad. Sci. USA* **107**, 17152–17157 (2010).

[34] Marks, J. *et al.* Context-specific inhibition of translation by ribosomal antibiotics targeting the peptidyl transferase center. *Proc. Nat. Acad. Sci. USA* **113**, 12150–12155 (2016).

[35] Choi, J. Dynamics of the context-specific translation arrest by chloramphenicol and linezolid. *Nat. Chem. Biol.* **16**, 310–317 (2019).

[36] Nakahigashi, K. *et al.* Comprehensive identification of translation start sites by tetracycline-inhibited ribosome profiling. *DNA Research* **23**, 193–201 (2016).

[37] Vazquez-Laslop, N. & Mankin, A. How macrolide antibiotics work. *Trends Biochem. Sci.* **43**, 668–684 (2018).

[38] Simms, C., Yan, L., Qiu, J. & Zaher, H. Ribosome collisions result in +1 frameshifting in the absence of no-go decay. *Cell Rep.* **28**, 1679–1689 (2019).

[39] Keiler, K. Mechanisms of ribosome rescue in bacteria. *Nat. Rev. Microbiol.* **13**, 285–297 (2015).

[40] Chadani, Y., Ono, K., Kutsukake, K. & Abo, T. *Escherichia coli* YaeJ protein mediates a novel ribosome-rescue pathway distinct from SsrA- and ArfA-mediated pathways. *Mol. Microbiol.* **80**, 772–785 (2011).

[41] Maaløe, O. *Biological Regulation and Development* (Plenum Press, New York, 1979).

[42] Gordon, J. Regulation of the in vivo synthesis of the polypeptide chain elongation factors in *Escherichia coli*. *Biochemistry* **9**, 912–917 (1970).

[43] Blumenthal, R., Lemaux, P., Neidhardt, F. & Dennis, P. The effects of the *relA* gene on the synthesis of aminoacyl-tRNA synthetases and other transcription and translation proteins in *Escherichia coli* B. *Molec. gen. Genet.* **149**, 291–296 (1976).

[44] Furano, A. & Wittel, F. Syntheses of elongation factors Tu and G are under stringent control in *Escherichia coli*. *J. Biol. Chem.* **251**, 898–901 (1976).

[45] Milon, P. *et al.* The nucleotide-binding site of bacterial translation initiation factor 2 (IF2) as a metabolic sensor. *Proc. Nat. Acad. Sci. USA* **103**, 13962–13967 (2006).
