# Supplementary material for: Mechanisms of drug interactions between translation-inhibiting antibiotics
Source: Nat Commun. 2020 Aug 11;11:4013. doi: 10.1038/s41467-020-17734-z (PMC7421507; doi:10.1038/s41467-020-17734-z)
Supplement: Supplementary file 3 — Description of Additional Supplementary Files [file 41467_2020_17734_MOESM3_ESM.pdf]

**Description of Additional Supplementary Files**

File Name: Supplementary Data 1

Description: **Oligonucleotides used in this study:** primer names, sequences, templates, and brief description of use. Each tab corresponds to the aim of a specific cloning step.
