# Supplementary material for: Mechanisms of drug interactions between translation-inhibiting antibiotics
Source: Nat Commun. 2020 Aug 11;11:4013. doi: 10.1038/s41467-020-17734-z (PMC7421507; doi:10.1038/s41467-020-17734-z)
Supplement: Supplementary file 5 — Reporting Summary [file 41467_2020_17734_MOESM5_ESM.pdf]

nature research

Corresponding author(s): Tobias Bollenbach

Last updated by author(s): Jul 8, 2020

Reporting Summary

Nature Research wishes to improve the reproducibility of the work that we publish. This form provides structure for consistency and transparency in reporting. For further information on Nature Research policies, see our Editorial Policies and the Editorial Policy Checklist.

Statistics

For all statistical analyses, confirm that the following items are present in the figure legend, table legend, main text, or Methods section.

n/a	Confirmed	
☐	☒	The exact sample size (*n*) for each experimental group/condition, given as a discrete number and unit of measurement
☐	☒	A statement on whether measurements were taken from distinct samples or whether the same sample was measured repeatedly
☐	☒	The statistical test(s) used AND whether they are one- or two-sided<br>*Only common tests should be described solely by name; describe more complex techniques in the Methods section.*
☐	☒	A description of all covariates tested
☐	☒	A description of any assumptions or corrections, such as tests of normality and adjustment for multiple comparisons
☐	☒	A full description of the statistical parameters including central tendency (e.g. means) or other basic estimates (e.g. regression coefficient) AND variation (e.g. standard deviation) or associated estimates of uncertainty (e.g. confidence intervals)
☐	☒	For null hypothesis testing, the test statistic (e.g. *F*, *t*, *r*) with confidence intervals, effect sizes, degrees of freedom and *P* value noted<br>*Give P values as exact values whenever suitable.*
☒	☐	For Bayesian analysis, information on the choice of priors and Markov chain Monte Carlo settings
☒	☐	For hierarchical and complex designs, identification of the appropriate level for tests and full reporting of outcomes
☐	☒	Estimates of effect sizes (e.g. Cohen's *d*, Pearson's *r*), indicating how they were calculated

Our web collection on statistics for biologists contains articles on many of the points above.

Software and code

Policy information about availability of computer code

Data collection	Tecan M1000, controlled by Tecan i-control© (v1.10.4), which upon entry of the measurement parameters operated independently. Script is provided at https://doi.org/10.15479/AT:ISTA:8097
Data analysis	Custom Mathematica© (Wolfram Research, 11.3) scripts used for data analysis and modeling. Scripts are provided at https://doi.org/10.15479/AT:ISTA:8097

For manuscripts utilizing custom algorithms or software that are central to the research but not yet described in published literature, software must be made available to editors and reviewers. We strongly encourage code deposition in a community repository (e.g. GitHub). See the Nature Research guidelines for submitting code & software for further information.

Data

Policy information about availability of data

All manuscripts must include a data availability statement. This statement should provide the following information, where applicable:

- Accession codes, unique identifiers, or web links for publicly available datasets
- A list of figures that have associated raw data
- A description of any restrictions on data availability

The source data underlying all figures in the main and supplementary text are provided as a Source Data File.

Field-specific reporting

Please select the one below that is the best fit for your research. If you are not sure, read the appropriate sections before making your selection.

☒ Life sciences ☐ Behavioural & social sciences ☐ Ecological, evolutionary & environmental sciences

For a reference copy of the document with all sections, see nature.com/documents/nr-reporting-summary-flat.pdf

Life sciences study design

All studies must disclose on these points even when the disclosure is negative.

Sample size	Based on previous experience [see Chevereau and Bollenbach, Mol. Syst. Biol. (2015), Bollenbach et al., Cell (2009)] with drug interaction measurements and used sample sizes in literature [see Hegreness et al., PNAS (2008), Chait et al., Nature (2007), Barbosa et al, PLoS Biology (2017)], spreading the drug-drug gradients over 192 different wells adequately describes the shape of the dose-response surface and the type of interaction. We further increased the number of measured points for the double-titration assay in which the gradient was spread over 24x24 wells. The sample size for plating (Supplementary Fig. 7) was determined using Schaechter et al. [Microbiology (1958)] as guidance.
Data exclusions	The relative growth rates used in the analysis were either above 0.1 or 0, while the interval between these values was excluded due to well-known technical difficulties of quantifying such low growth rates. This criterion is consistent with previously used cut-off values [see e.g., Chait et al., Nature (2007), Chevereau and Bollenbach, Mol. Syst. Biol. (2015)]. All used data is included in the Source Data File.
Replication	We verified the reproducibility of growth-rate measurements of drug interactions by calculating correlations between growth-rate measurements in the same condition on different days (Supplementary Fig. 1d). Similarly, we validated the reproducibility of Loewe interaction scores measured on different days (Supplementary Fig. 1f). The replicated measurements generally agreed well (Supplementary Fig. 1a). Dose-response curves of antibiotics (Supplementary Figs. 1g, 2a) and translation bottlenecks (Fig. 3c and Supplementary Fig. 3a) were measured in n=7 and n=8 replicates, respectively. We did not replicate the individual growth-rate measurements for drug-bottleneck interactions, as we only sought the general trend; the latter was based on multiple independent measurements. We verified the effect of individual measurements by bootstrapping. Verification of plasmid retention assessed by counting colony forming units was repeated in n=10 or n=8 technical replicates (as indicated in Supplementary Fig. 7b).
Randomization	We did not randomize the measurements among the wells on the plate, as we verified that the measurements are reproducible even if the general plate arrangement is altered (Supplementary Fig. 1d).
Blinding	In general, blinding was not applicable. We applied an agglomerative unsupervised method for clustering, which required no other input (such as the number of clusters) by the investigators apart from the data and the distance metric used.

Reporting for specific materials, systems and methods

We require information from authors about some types of materials, experimental systems and methods used in many studies. Here, indicate whether each material, system or method listed is relevant to your study. If you are not sure if a list item applies to your research, read the appropriate section before selecting a response.

Materials & experimental systems

n/a	Involved in the study
☒	☐ Antibodies
☒	☐ Eukaryotic cell lines
☒	☐ Palaeontology and archaeology
☒	☐ Animals and other organisms
☒	☐ Human research participants
☒	☐ Clinical data
☒	☐ Dual use research of concern

Methods

n/a	Involved in the study
☒	☐ ChIP-seq
☒	☐ Flow cytometry
☒	☐ MRI-based neuroimaging
