# Supplementary figures and images for: Mechanisms of drug interactions between translation-inhibiting antibiotics
Source: Nat Commun. 2020 Aug 11;11:4013. doi: 10.1038/s41467-020-17734-z (PMC7421507; doi:10.1038/s41467-020-17734-z)

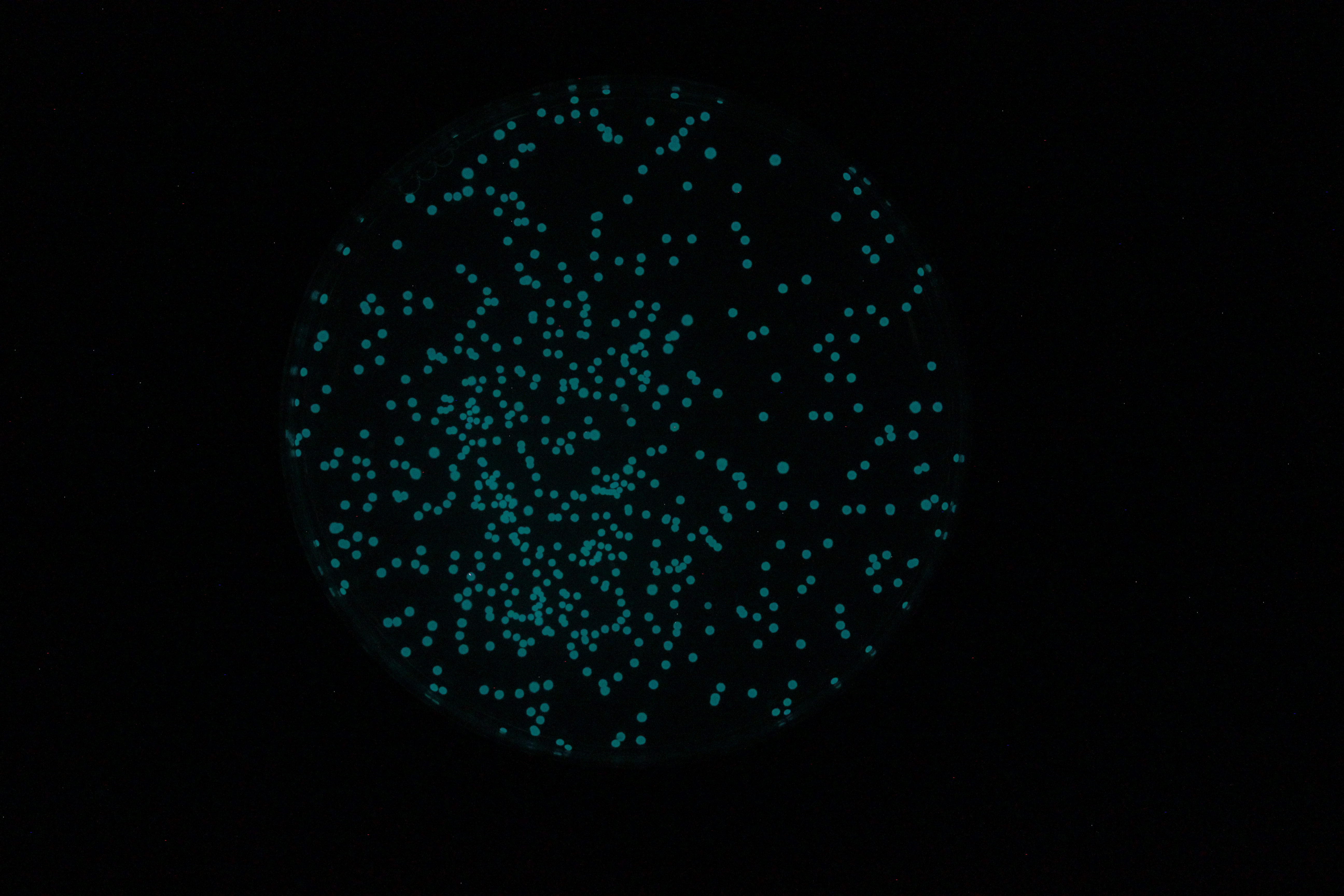

Supplement: Supplementary file 6 — Source Data [file 41467_2020_17734_MOESM6_ESM.zip › data_NatComm202006_finalSubmission/figS7/S7c_images/w1-chl-no flash.JPG]

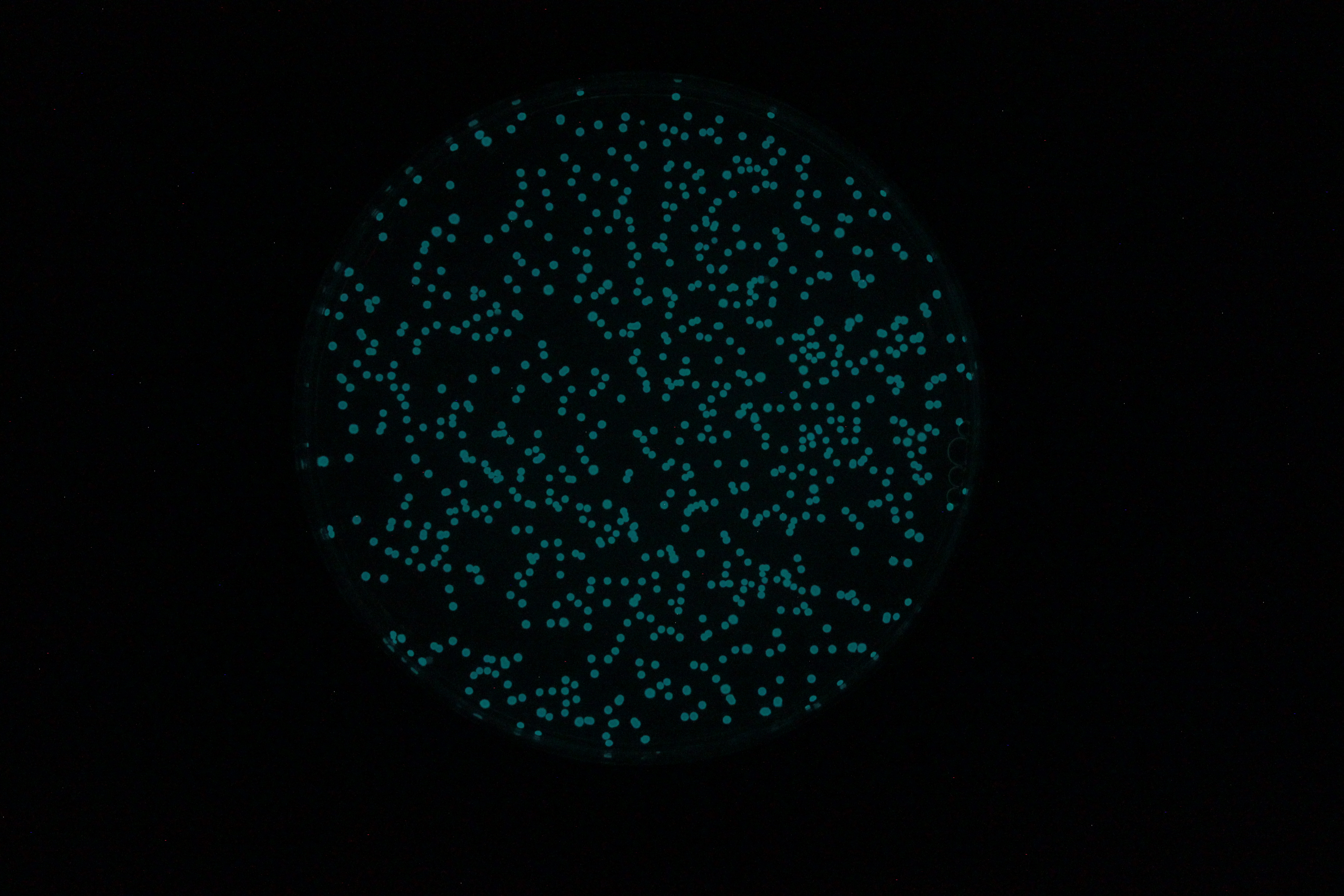

Supplement: Supplementary file 6 — Source Data [file 41467_2020_17734_MOESM6_ESM.zip › data_NatComm202006_finalSubmission/figS7/S7c_images/w2-kanchl-no flash.JPG]

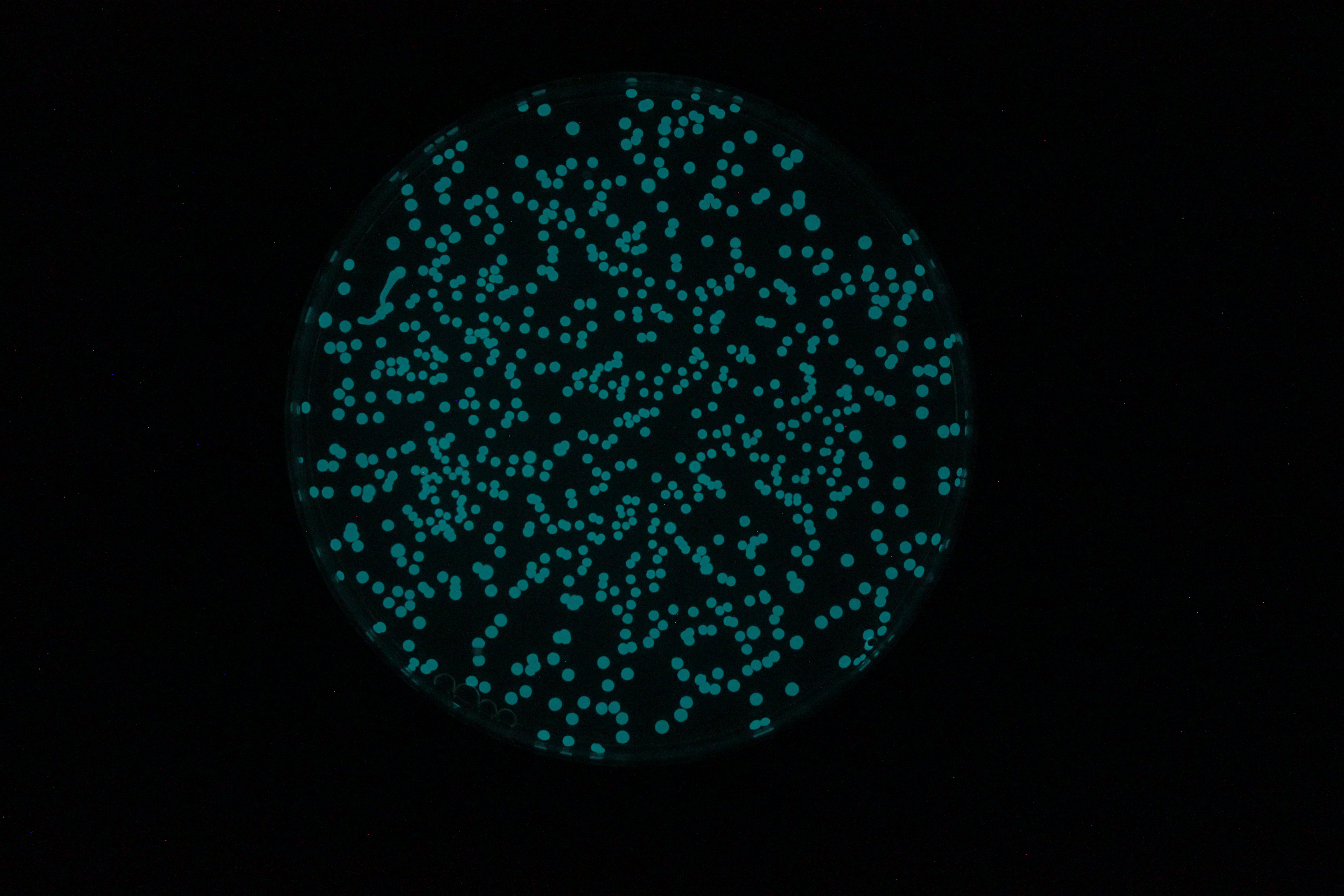

Supplement: Supplementary file 6 — Source Data [file 41467_2020_17734_MOESM6_ESM.zip › data_NatComm202006_finalSubmission/figS7/S7c_images/w-1-kan-no flash.JPG]

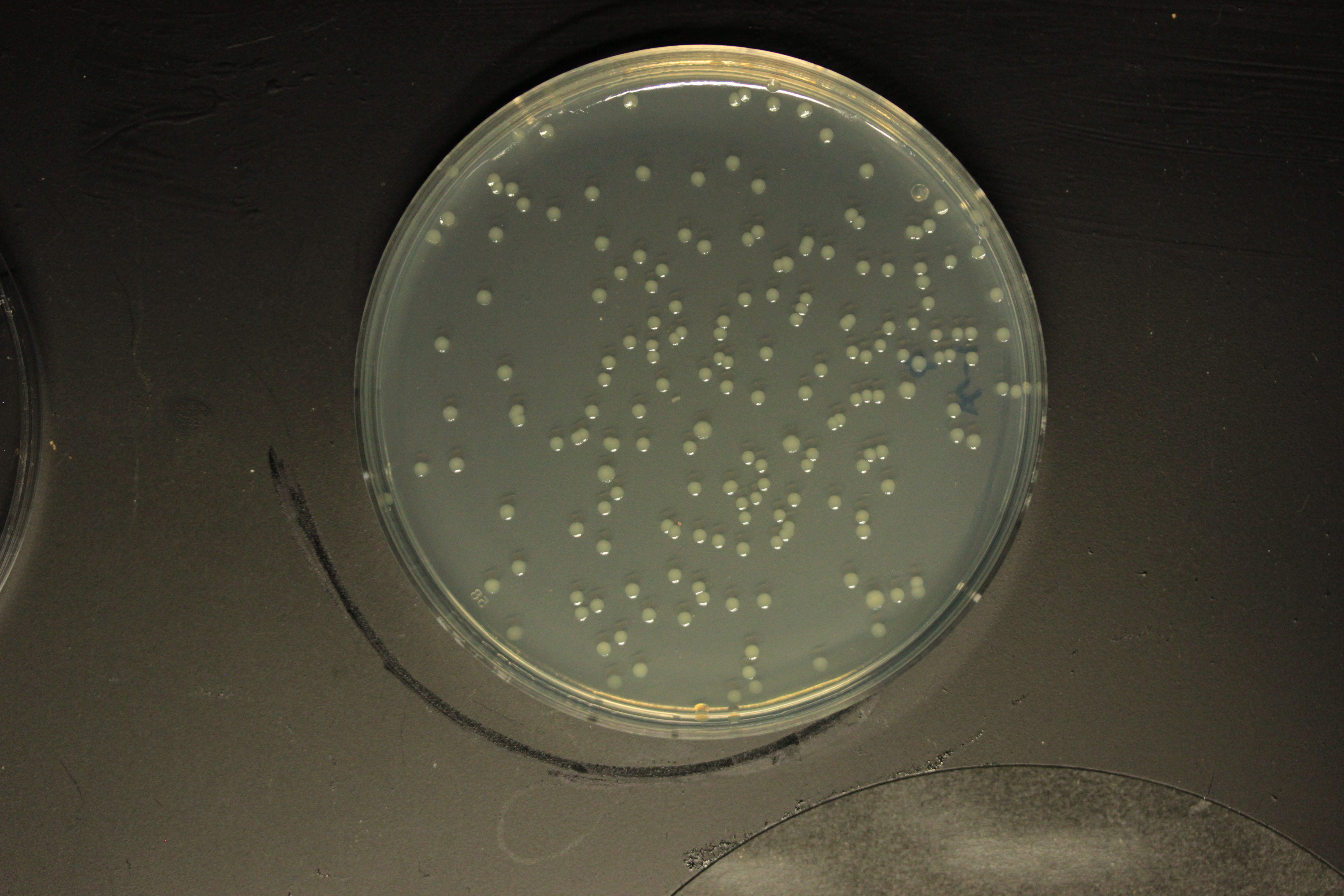

Supplement: Supplementary file 6 — Source Data [file 41467_2020_17734_MOESM6_ESM.zip › data_NatComm202006_finalSubmission/figS7/S7c_images/w1-0-bf.JPG]

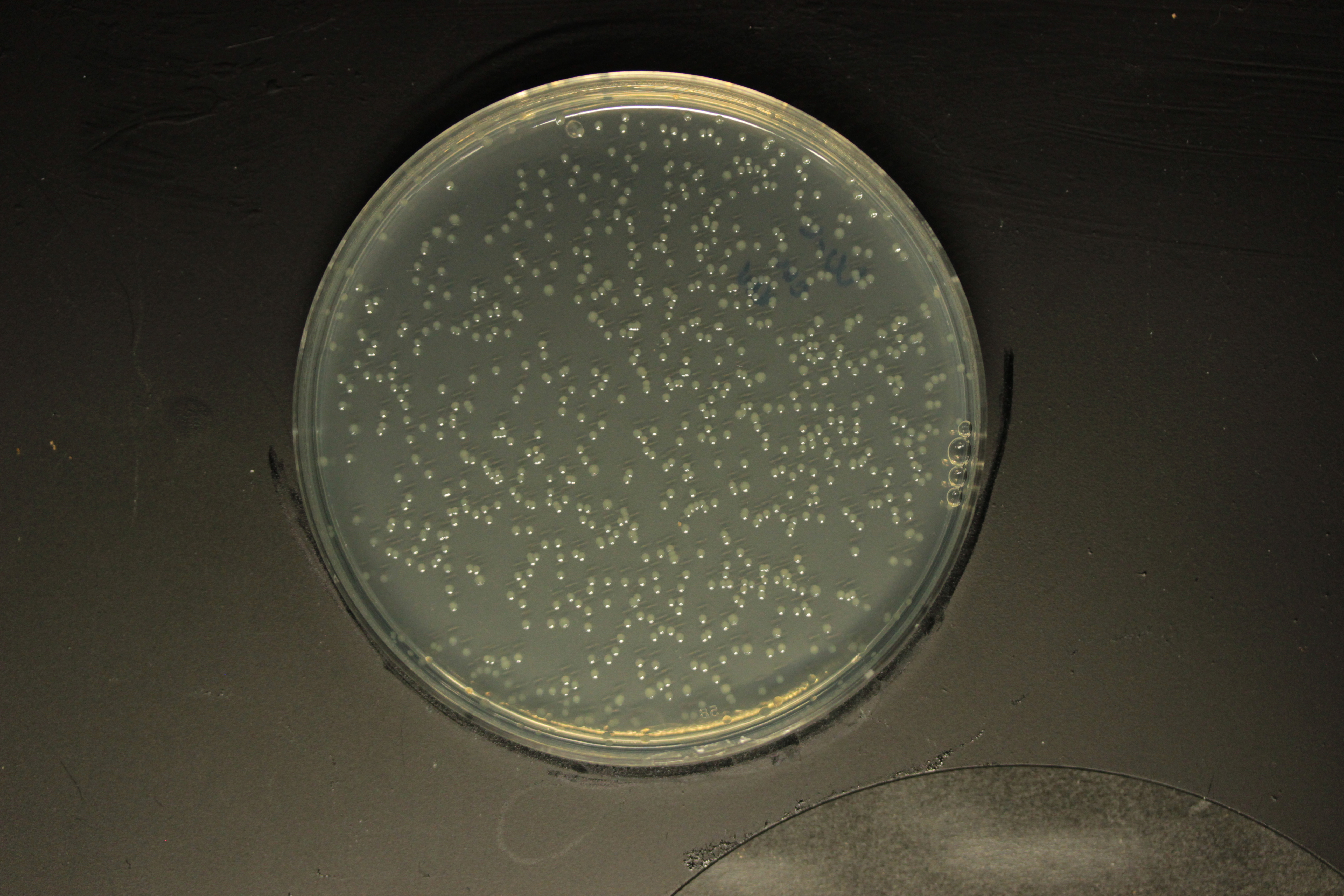

Supplement: Supplementary file 6 — Source Data [file 41467_2020_17734_MOESM6_ESM.zip › data_NatComm202006_finalSubmission/figS7/S7c_images/w2-kanchl-bf.JPG]

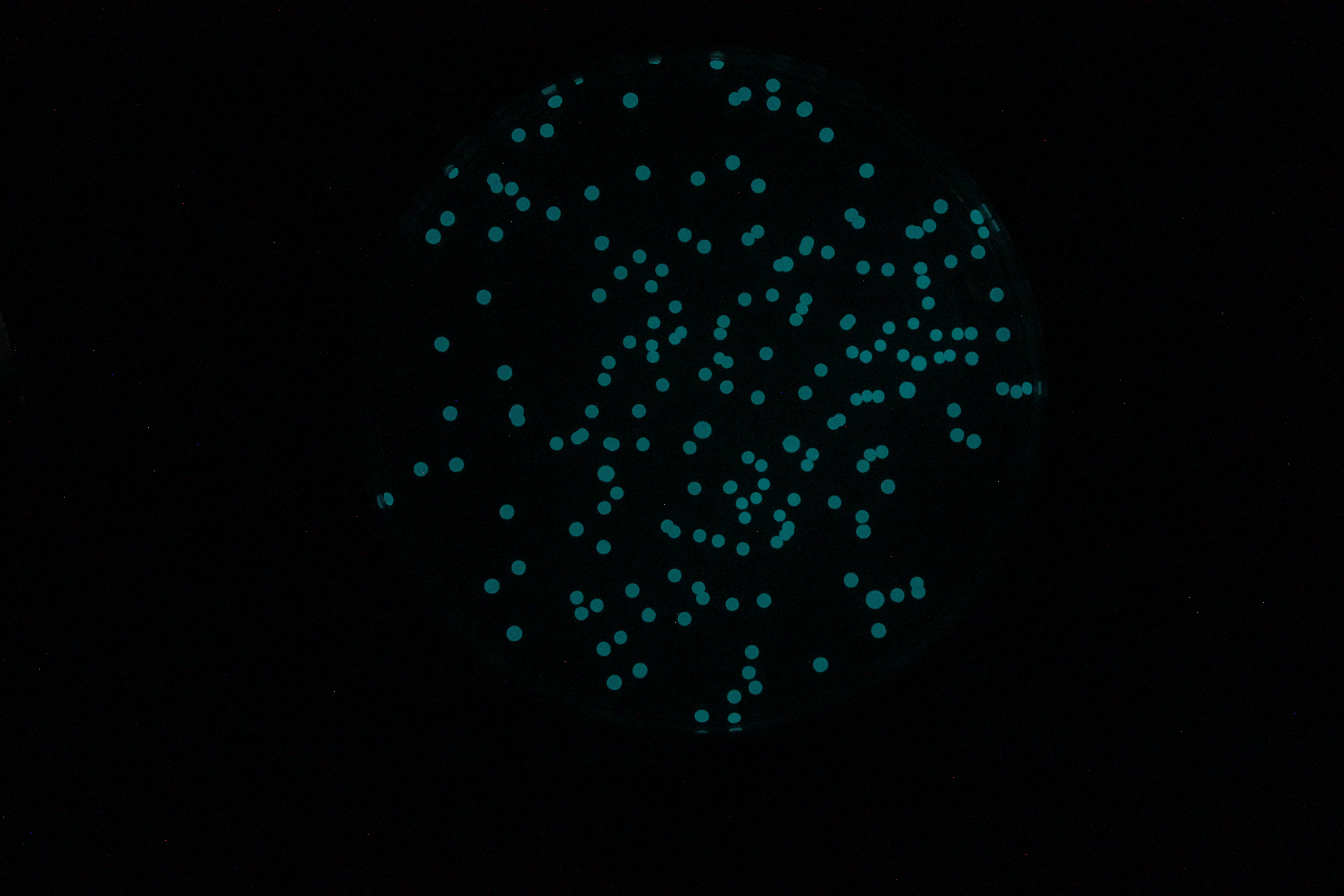

Supplement: Supplementary file 6 — Source Data [file 41467_2020_17734_MOESM6_ESM.zip › data_NatComm202006_finalSubmission/figS7/S7c_images/w1-0-no flash.JPG]

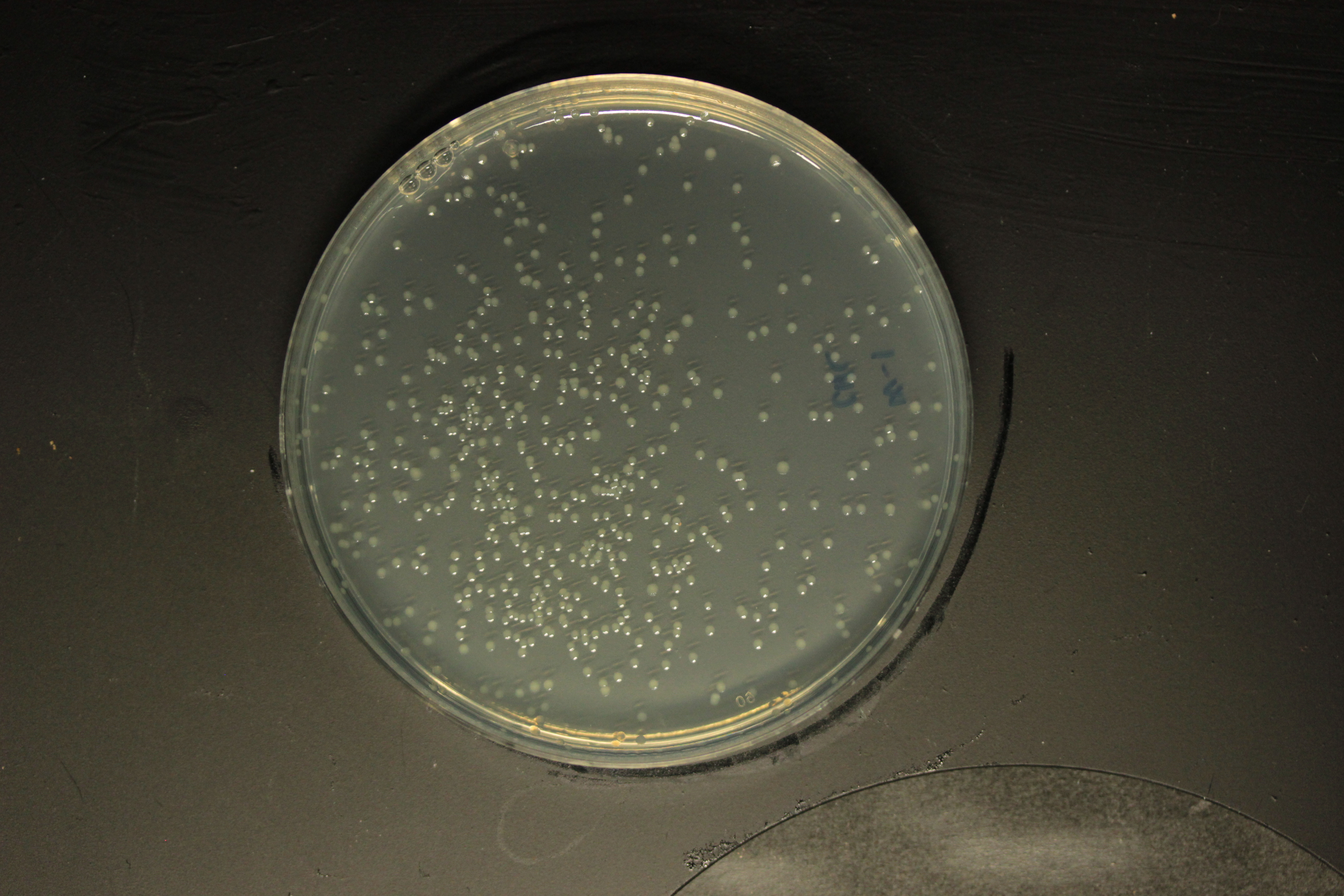

Supplement: Supplementary file 6 — Source Data [file 41467_2020_17734_MOESM6_ESM.zip › data_NatComm202006_finalSubmission/figS7/S7c_images/w1-chl-bf.JPG]

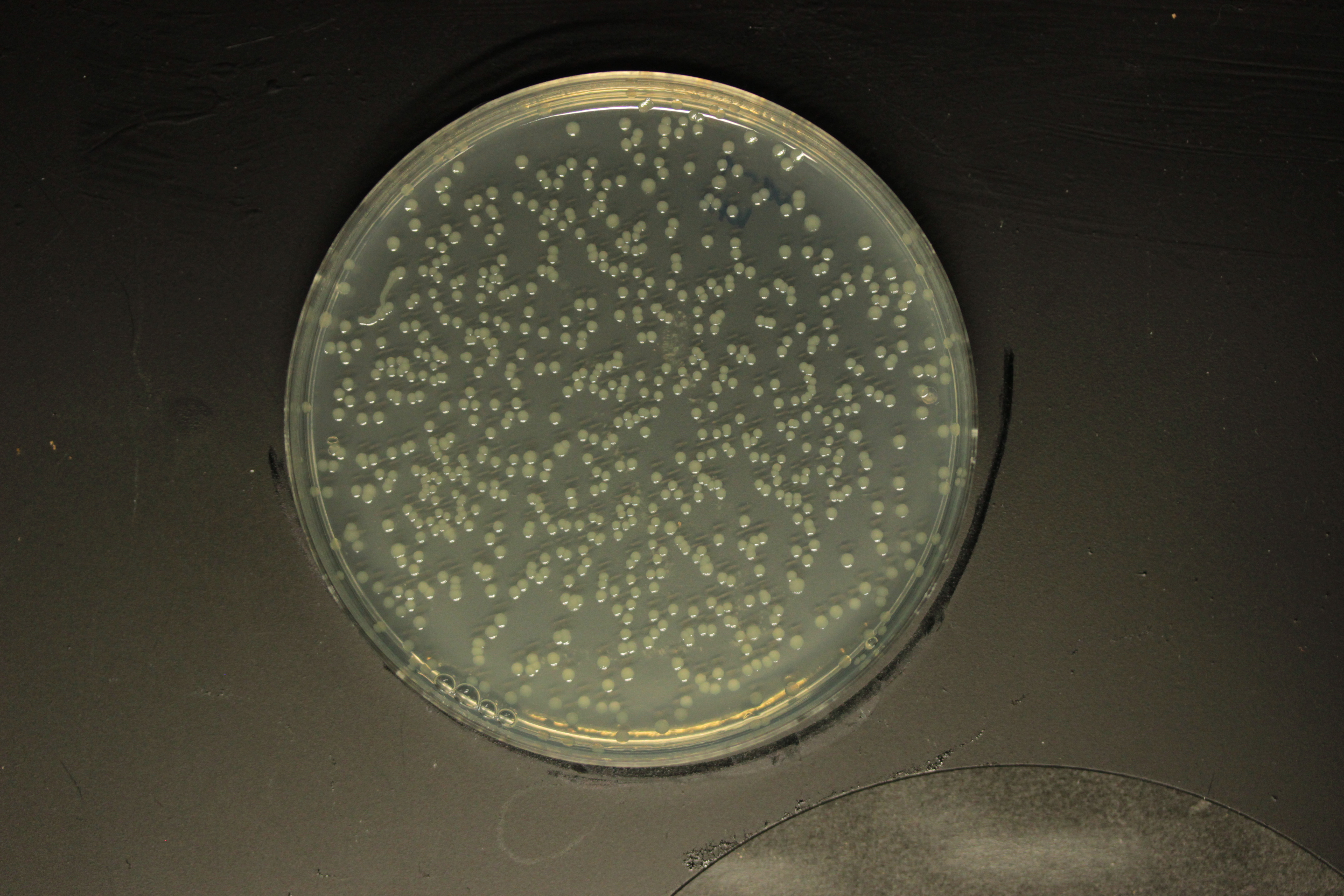

Supplement: Supplementary file 6 — Source Data [file 41467_2020_17734_MOESM6_ESM.zip › data_NatComm202006_finalSubmission/figS7/S7c_images/w-1-kan-bf.JPG]

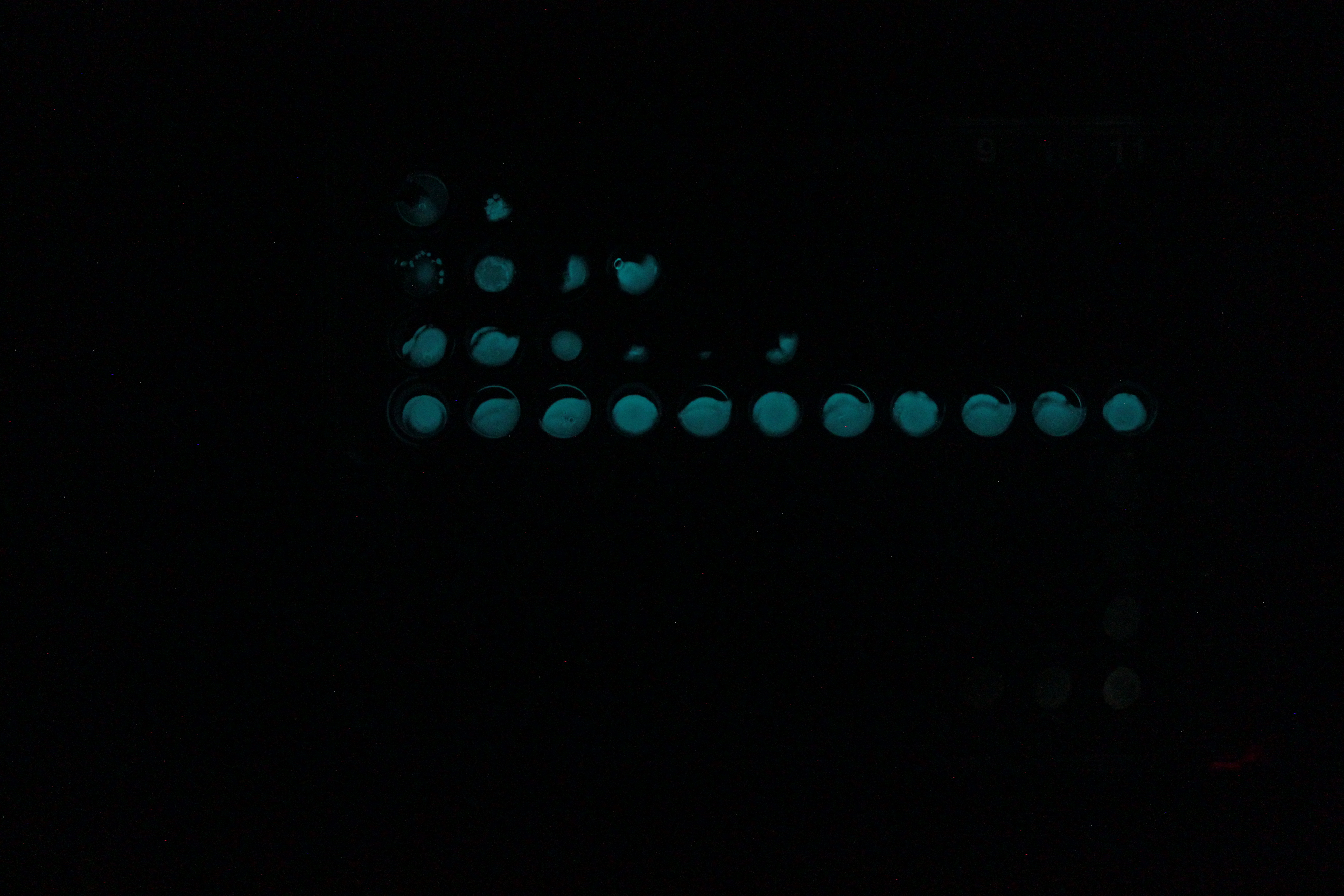

Supplement: Supplementary file 6 — Source Data [file 41467_2020_17734_MOESM6_ESM.zip › data_NatComm202006_finalSubmission/fig6/images_6b/plate-luminescence_00-10-01-11.JPG]

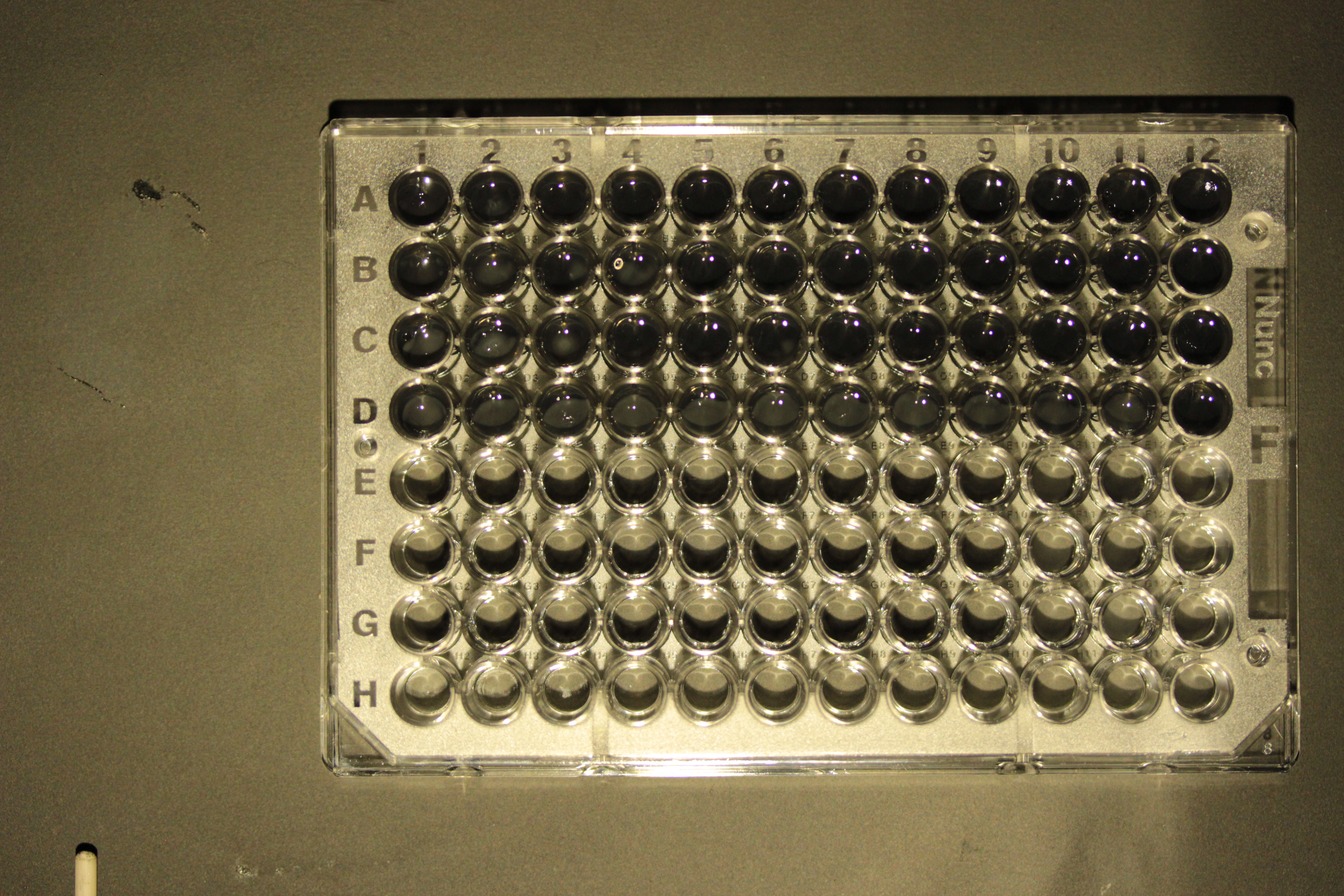

Supplement: Supplementary file 6 — Source Data [file 41467_2020_17734_MOESM6_ESM.zip › data_NatComm202006_finalSubmission/fig6/images_6b/plate-bright-field_00-10-01-11.jpg]
